# Caspase-10 inhibits ATP-citrate lyase-mediated metabolic and epigenetic reprogramming to suppress tumorigenesis

**DOI:** 10.1038/s41467-019-12194-6

**Published:** 2019-09-18

**Authors:** Rajni Kumari, Ruhi S. Deshmukh, Sanjeev Das

**Affiliations:** Molecular Oncology Laboratory, National Institute of Immunology, Aruna Asaf Ali Marg, New Delhi, 110067 India

**Keywords:** Cancer, Cancer metabolism, Tumour-suppressor proteins, Epigenetics

## Abstract

Caspase-10 belongs to the class of initiator caspases and is a close homolog of caspase-8. However, the lack of caspase-10 in mice and limited substrate repertoire restricts the understanding of its physiological functions. Here, we report that ATP-citrate lyase (ACLY) is a caspase-10 substrate. Caspase-10 cleaves ACLY at the conserved Asp1026 site under conditions of altered metabolic homeostasis. Cleavage of ACLY abrogates its enzymatic activity and suppresses the generation of acetyl-CoA, which is critical for lipogenesis and histone acetylation. Thus, caspase-10-mediated ACLY cleavage results in reduced intracellular lipid levels and represses GCN5-mediated histone H3 and H4 acetylation. Furthermore, decline in GCN5 activity alters the epigenetic profile, resulting in downregulation of proliferative and metastatic genes. Thus caspase-10 suppresses ACLY-promoted malignant phenotype. These findings expand the substrate repertoire of caspase-10 and highlight its pivotal role in inhibiting tumorigenesis through metabolic and epigenetic mechanisms.

## Abstract (web summary)

Caspases are most closely associated with cell death, but many have other cellular functions. Here, Das et al. find that upon metabolic stress, caspase-10 cleaves ACLY to regulate metabolic homeostasis and epigenetic reprogramming by altering Acetyl-CoA levels.

## Introduction

Caspase-10 is a close homolog of caspase-8^1^. By virtue of its structural similarity with caspase-8, caspase-10 has been reported to mediate extrinsic pathway of apoptosis by binding to death-induced silencing complex (DISC)^2^. Owing to its role in apoptosis, loss-of-function mutations in caspase-10 have been reported in several cancers including non-Hodgkin lymphoma, gastric carcinoma, and non-small cell lung carcinoma^3–5^. Epigenetic silencing of caspase-10, has also been reported in pediatric tumors, acute promyelocytic leukemia and colorectal cancer^6–8^. Besides apoptosis, caspases have also been observed to regulate other cellular processes including metabolic pathways. Caspase-1 has been reported to regulate glycolysis by cleaving glyceraldehyde-3-phosphate dehydrogenase in macrophages^9^. Under metabolic stress conditions, caspase-3 has been shown to cleave the common α-subunit of farnesyltransferase and geranylgeranyltransferase and regulate insulin secretion^10^. Since little is known about caspase-10 substrates, its role in pathways apart from apoptosis remains unexplored.

ATP-citrate lyase (ACLY) is a cytosolic enzyme which catalyzes the conversion of citrate to acetyl-CoA and oxaloacetate. This reaction is critical for maintaining the pool of acetyl-CoA in cytoplasm and nucleus, which is the building block for lipogenesis and protein acetylation, including histones^11,12^. Lipogenesis and epigenetic reprogramming are key drivers of tumorigenesis^13,14^. Thus ACLY levels and activity are upregulated by oncogenic drivers including PI3K-Akt pathway^15,16^. Furthermore, increased expression and elevated activity of ACLY has been reported in several cancers including non-small cell lung carcinoma^11,16^.

In this study, we report that ACLY is a novel caspase-10 substrate. Cleavage of ACLY depletes the cellular acetyl-CoA pool which leads to suppression of lipogenesis and GCN5-mediated epigenetic regulation of proliferative and metastatic genes. Thus caspase-10 inhibits ACLY promoted tumorigenesis.

## Results

### Caspase-10 is upregulated upon metabolic stress

**Fig. 1:**
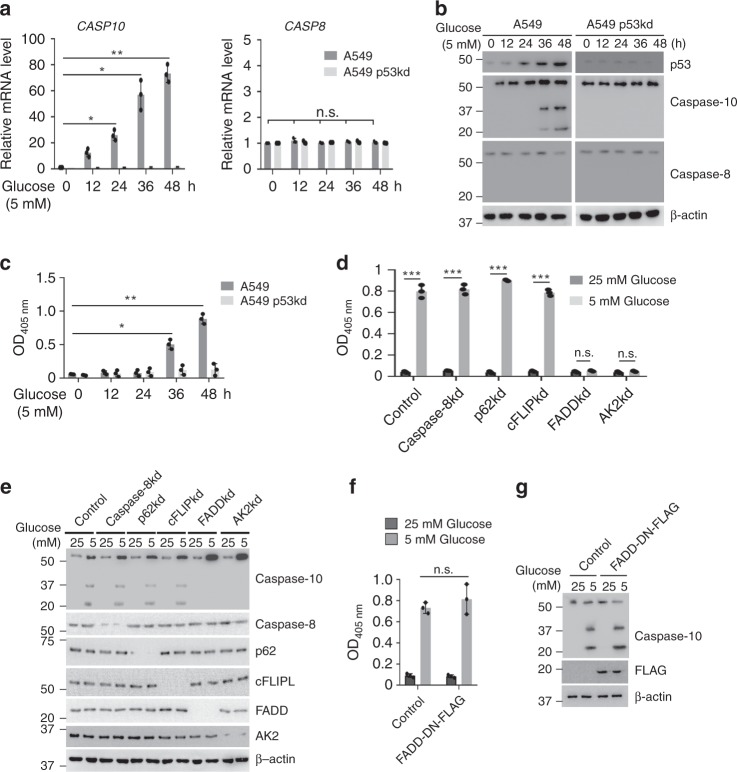
Caspase-10 is upregulated upon metabolic stress **a** A549 cells were stably transfected (pooled puromycin-resistant population) with control (scrambled) or p53 shRNA. A549 control (A549) and p53 knockdown (A549 p53kd) cells were subjected to glucose starvation for the indicated time points. Relative mRNA levels were analyzed by RT-qPCR for the indicated genes. Error bars are means ± SD of three biological replicates. Statistical analyses were done using two-way ANOVA (Tukey’s post hoc test). *P < 0.05; **P < 0.01. **b** A549 control (A549) and p53 knockdown (A549 p53kd) cells were subjected to glucose starvation for the indicated time points. The cells were then harvested and western blotting was performed for indicated proteins. **c** A549 control (A549) and A549 p53 knockdown (A549 p53kd) cells were subjected to glucose starvation for the indicated time points. The cells were then harvested and caspase-10 activity was examined. Error bars are means ± SD of three biological replicates. Statistical analyses were done using two-way ANOVA (Tukey’s post hoc test). *P < 0.05; **P < 0.01. **d**, **e** A549 cells were stably transfected (pooled puromycin-resistant population) with control (LacZ) or the indicated shRNAs. Cells were subjected to unstressed (25 mM) or glucose starvation conditions (5 mM) for 36 h. The cells were then harvested and, (**d**) caspase-10 activity was examined or (**e**) western blotting was performed for the indicated proteins. Error bars are means ± SD of three biological replicates. Statistical analyses were done using two-way ANOVA (Bonferroni’s post hoc test). ***P < 0.001. **f**, **g** A549 cells were stably transfected with either empty vector or dominant negative mutant FADD-DN-FLAG construct. These cells were subjected to unstressed (25 mM) or glucose starvation conditions (5 mM) for 36 h. The cells were then harvested and (**f**) caspase-10 activity was examined or (**g**) western blotting was performed for the indicated proteins. Error bars are means ± SD of three biological replicates. Statistical analyses were done using two-way ANOVA (Bonferroni’s post hoc test). Source data are provided as a Source Data file

Previously, p53 has been reported to upregulate caspase-10 in response to genotoxic stress^17^. To investigate whether caspase-10 was regulated by p53 under metabolic stress conditions, we examined caspase-10 levels in the presence or absence of p53 under metabolic stress conditions including glucose starvation, serum deprivation and metformin treatment. We observed a significant increase in caspase-10 levels over the time course of metabolic stress (Fig. 1a, b, Supplementary Fig. 1 a–d). However, induction of caspase-10 was abrogated in the absence of p53. Similar results were obtained in other cell types, including HCT116 and IMR90 (Supplementary Fig. 1 e, f). Moreover, there was no change in the levels of caspase-10 homolog caspase-8 which suggests that caspase-10 is specifically upregulated upon metabolic stress in a p53-dependent manner. We also observed cleavage of caspase-10 following its upregulation at 36 h and 48 h of metabolic stress, indicative of homodimerization-induced autocleavage of caspase-10. Hence, we further examined caspase-10 activity upon metabolic stress. We observed enhanced activity of caspase-10 at 36 h and 48 h of metabolic stress, while in the absence of p53, limited caspase-10 activity was detected (Fig. 1c). Altogether, these results indicate that caspase-10 is upregulated and activated under metabolic stress conditions in a p53-dependent manner.

Since the activation of caspase-10 is associated with the induction of apoptosis^18^, we also examined the kinetics of induction of apoptosis under metabolic stress conditions. We observed that there was significant apoptosis only upon prolonged glucose starvation (72 h) (Supplementary Fig. 1g). To delineate the mode of caspase-10 activation under metabolic stress conditions, we depleted previously reported oligomerization-inducing partners of caspase-10 including caspase-8, cFLIP_L_, FADD, AK2, and p62^18–21^ under glucose starvation conditions. We observed that the depletion of FADD or AK2 led to the inhibition of caspase-10 activity and autocleavage of caspase-10 under metabolic stress conditions (Fig. 1d, e). To further examine whether the involvement of FADD is mediated by death-receptors, we ectopically expressed dominant-negative mutant of FADD (FADD-DN) which has been reported to inhibit death receptor-mediated signaling^22^. No significant change in caspase-10 activation or cleavage was observed in presence of FADD-DN (Fig. 1f, g). Taken together, these results suggest that AK2 and FADD are crucial for caspase-10 activation upon metabolic stress and this activation is independent of death-receptors. In addition, depletion of major effector caspase, caspase-3 had no effect on caspase-10 activation under metabolic stress conditions (Supplementary Fig. 1h). The time lag between the kinetics of caspase-10 activation upon metabolic stress and induction of apoptosis suggests possible non-apoptotic functions of caspase-10.

### Caspase-10 cleaves ACLY

**Fig. 2:**
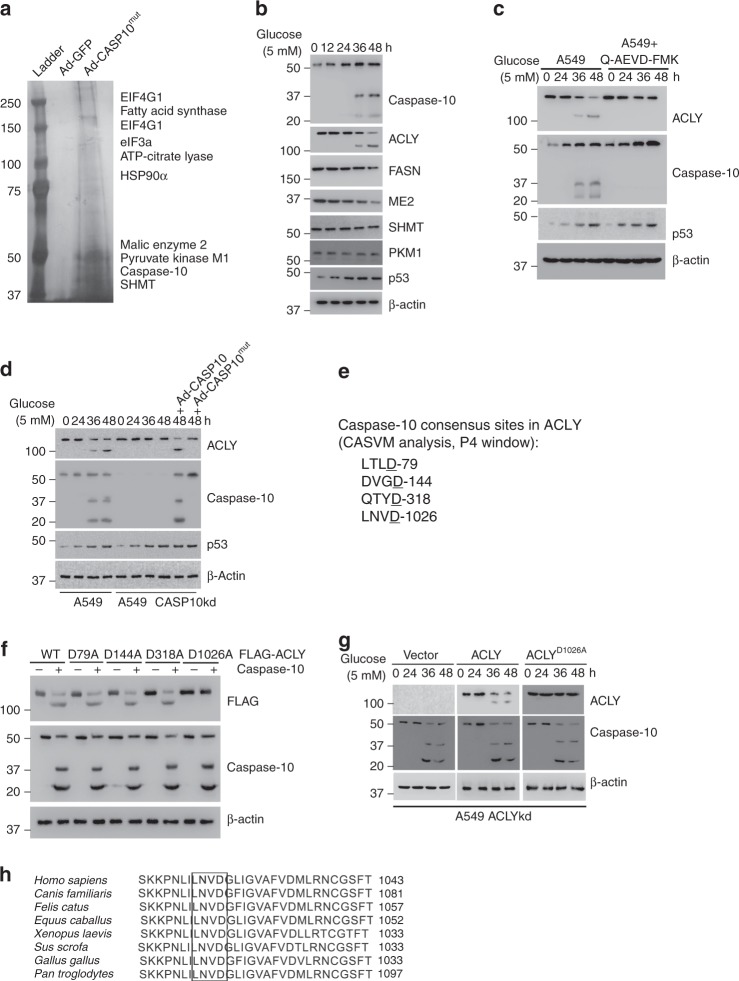
Caspase-10 cleaves ATP-citrate lyase **a** H1299 cells were infected with adenovirus expressing GFP (Ad-GFP) or caspase-10^mut^ tagged with FLAG and HA epitopes (Ad-CASP10^mut^). Twenty-four hours post-infection, cell extracts were sequentially immunoprecipitated with FLAG and HA antibody affinity resins. The caspase-10 associated proteins were detected by SDS-PAGE and silver staining. **b** A549 cells were subjected to glucose starvation for the indicated time points. The cells were then harvested and western blotting was performed for the indicated proteins. **c** A549 cells were subjected to glucose starvation and concomitantly treated with DMSO (control) or caspase-10 inhibitor (Q-AEVD-FMK) (25 μM) for the indicated time points. The cells were then harvested, and western blotting was performed for the indicated proteins. **d** A549 cells were stably transfected (pooled zeocin-resistant population) with control (scrambled) or caspase-10 shRNA. A549 control (A549) and caspase-10 knockdown (A549 CASP10kd) cells were subjected to glucose starvation for the indicated time points. A549 CASP10kd cells were infected with adenovirus expressing wild-type caspase-10 (Ad-CASP10) or catalytically dead caspase-10 mutant (Ad-CASP10^mut^) during the last 18 h prior to the end of the 48 h time point, as indicated. The cells were then harvested and western blotting was performed for the indicated proteins. **e** Predicted caspase-10-targeted aspartate residue(s) of ACLY from CASVM analysis. **f** A549 cells were co-transfected with caspase-10 and FLAG-tagged wild-type ACLY or mutants as indicated. 24 h post-transfection, the cells were harvested and western blotting was performed for the indicated proteins. **g** A549 cells were stably transfected (pooled zeomycin-resistant population) with ACLY shRNA. A549 ACLY knockdown cells (A549 ACLYkd) were stably transfected (pooled hygromycin-resistant population) with empty vector, wild-type (ACLY) or mutant ACLY (ACLY^D1026A^). These cells were subjected to glucose starvation for the indicated time points followed by western blotting for the indicated proteins. **h** Sequence alignment of ACLY for caspase-10 targeted site in different organisms. Source data are provided as a Source Data file

To explore the functions of caspase-10 upon metabolic stress, we employed a proteomics approach to identify caspase-10 interacting proteins (Fig. 2a). Since ectopic expression of wild-type caspase-10 triggers apoptosis, a protease-dead mutant of caspase-10 (CASP10^C401A^)^23^ was used for the proteomics screen. Amongst the novel interactors, metabolic enzymes viz. FASN (Fatty acid synthase), ACLY (ATP-citrate lyase), PKM1 (Pyruvate kinase M1), SHMT (Serine hydroxymethyltransferase), and ME2 (Malic enzyme 2) were of particular interest as our earlier results indicated that caspases-10 was upregulated and activated upon metabolic stress. Hence, we examined the status of these metabolic enzymes upon caspase-10 upregulation and activation under glucose starvation conditions. We observed that ACLY was the only enzyme cleaved under glucose starvation conditions (Fig. 2b). We further validated our proteomics screen findings by performing in vitro binding assay using recombinant CASP10^C401A^ mutant and ACLY proteins (Supplementary Fig. 2a). To corroborate a role for caspase-10 in ACLY cleavage, we performed in vitro cleavage assay. Our results indicated that caspase-10 can cleave ACLY (Supplementary Fig. 2b). We further examined ACLY status upon metabolic stress in the presence or absence of caspase-10 inhibitor, Q-AEVD-FMK. We observed that concomitant to gradual upregulation and activation of caspase-10, the cleavage of ACLY also gradually increased at extended periods of metabolic stress (36 h and 48 h). However, no cleavage of ACLY was observed upon Q-AEVD-FMK treatment (Fig. 2c). To rule out the non-specific inhibition of other caspases by Q-AEVD-FMK, we next examined the cleavage of ACLY upon metabolic stress in the presence or absence of caspase-10. We observed that depletion of caspase-10 abrogated ACLY cleavage under metabolic stress conditions. Moreover, ectopic expression of wild-type caspase-10, but not the protease-dead mutant, restored the cleavage of ACLY at extended periods of metabolic stress (Fig. 2d, Supplementary Fig. 2c). Similar results were obtained in other cell types, including HCT116 and IMR90 (Supplementary Fig. 2d, e).

Next, to identify the putative aspartate residue in ACLY which is targeted by caspase-10, we analyzed the protein sequence of ACLY in silico for caspase-10 consensus sites using CASVM online server^24^. Four aspartate residues (D79, D144, D318, and D1026) were predicted to be targeted by caspase-10 (Fig. 2e). To identify which of these aspartate residues is targeted by caspase-10, we performed site-directed mutagenesis. We observed that mutation of ACLY at D79, D144, and D318 did not inhibit the caspase-10-mediated cleavage. However, mutation of ACLY at D1026 abrogated the caspase-10-mediated cleavage even though the interaction could still be observed (Fig. 2f, Supplementary Fig. 2f). These results indicated that ACLY could be cleaved at D1026 upon metabolic stress. We further observed that, unlike wild-type ACLY, ACLY^D1026A^ was resistant to caspase-10-mediated cleavage under metabolic stress conditions (Fig. 2g, Supplementary Fig. 2g, h). Similar results were obtained in other cell types, such as HCT116 and IMR90 (Supplementary Fig. 2i, j). Moreover, sequence alignment of ACLY protein sequence revealed that caspase-10 targeting sequence (LNVD) is conserved across diverse species expressing caspase-10 (Fig. 2h). Altogether, our results demonstrate that ACLY is a caspase-10 substrate and is cleaved at D1026 site upon metabolic stress.

### Caspase-10 determines nucleocytosolic acetyl-CoA pool

**Fig. 3:**
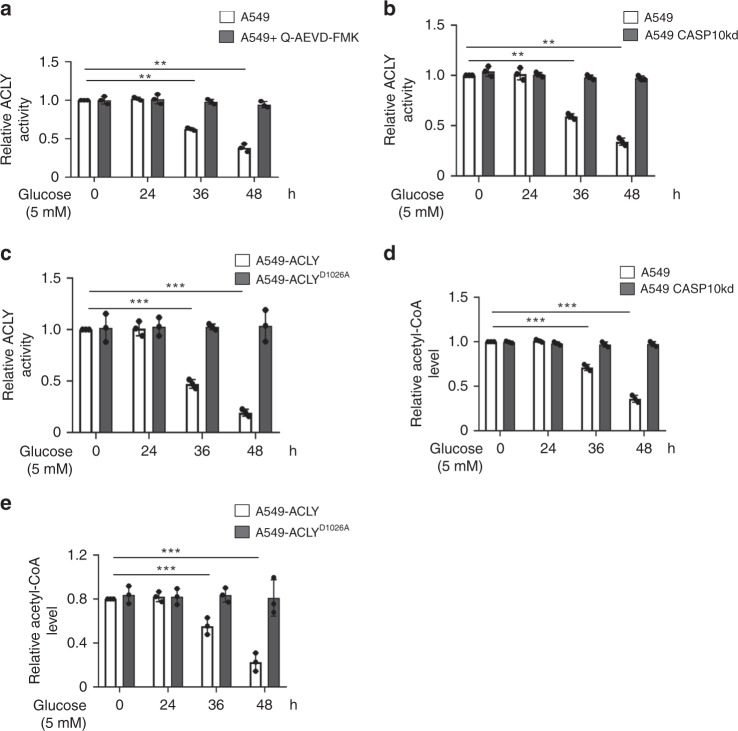
Caspase-10 regulates nucleocytosolic acetyl-CoA levels **a** A549 cells were subjected to glucose starvation and concomitantly treated with DMSO (control) or caspase-10 inhibitor (Q-AEVD-FMK) (25 μM) for the indicated time points. The cells were then harvested and ACLY activity was examined. Error bars are means ± SD of three biological replicates. Statistical analyses were done using two-way ANOVA (Bonferroni’s post hoc test). **P < 0.01. **b** A549 control (A549) and caspase-10 knockdown (A549 CASP10kd) cells were subjected to glucose starvation for the indicated time points. The cells were then harvested and ACLY activity was examined. Error bars are means ± SD of three biological replicates. Statistical analyses were done using two-way ANOVA (Bonferroni’s post hoc test). **P < 0.01. **c** A549 ACLY knockdown **c**ells (A549 ACLYkd) were stably transfected with wild-type ACLY (ACLY) or caspase-10-cleavage resistant mutant (ACLY^D1026A^). These cells were subjected to glucose starvation for the indicated time points followed by ACLY activity assay. Error bars are means ± SD of three biological replicates. Statistical analyses were done using two-way ANOVA (Bonferroni’s post hoc test). ***P < 0.001. **d** A549 control (A549) cells and A549 caspase-10 knockdown (A549 CASP10kd) cells were subjected to glucose starvation for the indicated time points. Nucleocytosolic fraction was then isolated followed by acetyl-CoA quantification. Error bars are means ± SD of three biological replicates. Statistical analyses were done using two-way ANOVA (Bonferroni’s post hoc test). ***P *<* 0.001. **e** A549 ACLY knockdown cells (A549 ACLYkd) w**e**re stably transfected with wild-type ACLY (ACLY) or caspase-10-cleavage resistant mutant (ACLY^D1026A^). These cells were subjected to glucose starvation for the indicated time points. Nucleocytosolic fraction was then isolated followed by acetyl-CoA quantification. Error bars are means ± SD of three biological replicates. Statistical analyses were done using two-way ANOVA (Bonferroni’s post hoc test). ***P *<* 0.001. Source data are provided as a Source Data file

We next investigated whether caspase-10-mediated cleavage of ACLY under metabolic stress conditions alters its enzymatic activity. We observed that enzymatic activity of ACLY declined at extended time periods of metabolic stress (36 h and 48 h). However, there was no significant effect on ACLY activity in presence of caspase-10 inhibitor Q-AEVD-FMK or upon caspase-10 depletion, under metabolic stress conditions (Fig. 3a, b, Supplementary Fig. 3a, b). Similar results were obtained in other cell types (Supplementary Fig. 3c, d). Furthermore, we examined the activity of cleavage-resistant mutant ACLY^D1026A^. The activity of wild-type ACLY, but not cleavage-resistant mutant ACLY^D1026A^ declined at extended time periods (36 h and 48 h) of metabolic stress (Fig. 3c). These findings suggest that metabolic-stress induced caspase-10-mediated cleavage of ACLY abrogates its activity.

ACLY catalyzes the conversion of cytosolic citrate to oxaloacetate and acetyl-CoA, which maintains acetyl-CoA homeostasis in nucleus and cytosol^11^. Hence, to further examine the effect of caspase-10 on ACLY activity, we examined acetyl-CoA levels. We observed a significant decline in nucleocytosolic acetyl-CoA levels at extended periods of metabolic stress (36 h and 48 h) (Fig. 3d, Supplementary Fig. 3e, f). However, we did not observe any significant change in nucleocytosolic acetyl-CoA levels in the absence of caspase-10 over the time course of metabolic stress. Similar results were obtained in other cell types (Supplementary Fig. 3g, h). Moreover, we observed that in presence of wild-type ACLY, acetyl-CoA levels declined at 36 h and 48 h of metabolic stress but there was no significant change in presence of cleavage-resistant mutant ACLY^D1026A^ (Fig. 3e). Taken together, these results indicate that caspase-10 regulates ACLY-mediated maintenance of acetyl-CoA levels.

### Caspase-10 regulates lipogenesis and histone acetylation

**Fig. 4:**
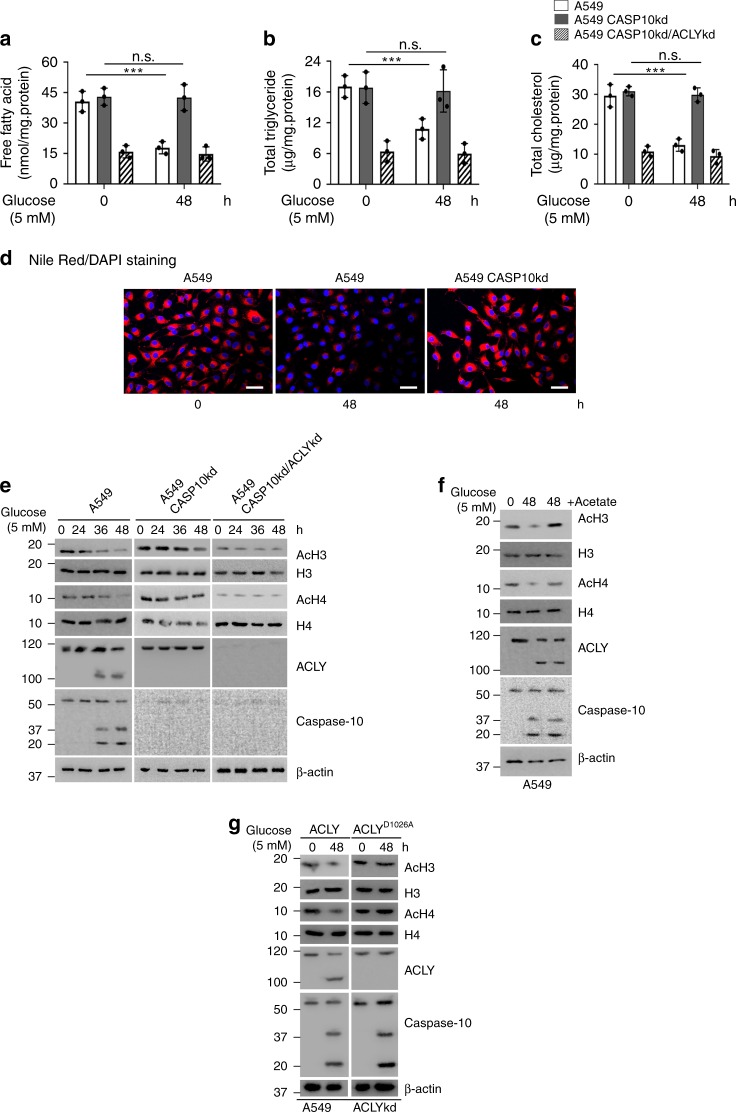
Caspase-10 downregulates intracellular lipid levels and histone acetylation **a**–**c** A549 cells were stably transfected (pooled zeocin-resistant population) with control (scrambled), caspase-10 shRNA or caspase-10 shRNA along with ACLY shRNA. A549 control (A549), CASP10 knockdown (A549 CASP10kd) and CASP10/ACLY double knockdown (A549 CASP10kd/ACLYkd) cells were subjected to glucose starvation for the indicated time points followed by (**a**) free fatty acids quantification, (**b**) total triglycerides quantification, and (**c**) total cholesterol quantification. Error bars are means ± SD of three biological replicates. Statistical analyses were done using two-way ANOVA (Bonferroni’s post hoc test). ***P < 0.001. **d** A549 cells were subjected to glucose starvation for 48 h and treated with caspase-10 inhibitor, Q-AEVD-FMK (25 μM) or palmitate (0.2 mM) for last 24 h of starvation period. The cells were then stained with Nile Red (5 μg/ml) and examined. DAPI was used to counterstain the nucleus. Scale bar: 50 μm. **e** A549 control (A549), caspase-10 knockdown (A549 CASP10kd) and caspase-10/ACLY double knockdown (A549 CASP10kd/ACLYkd) cells were subjected to glucose starvation for the indicated time points. The cells were then harvested and western blotting was performed for the indicated proteins. **f** A549 cells were subjected to glucose starvation for 48 h and treated with sodium acetate (5 mM) for the last 24 h of starvation period. The cells were then harvested and western blotting was performed for the indicated proteins. **g** A549 ACLY knockdown cells (A549 ACLYkd) were stably transfected with wild-type ACLY (ACLY) or caspase-10-cleavage resistant mutant (ACLY^D1026A^). These cells were subjected to glucose starvation for the indicated time points. The cells were then harvested and western blotting was performed for the indicated proteins. Source data are provided as a Source Data file

Since acetyl-CoA is the building block for lipogenesis, we investigated whether caspase-10 alters the levels of intracellular lipids viz. free fatty acids, triglycerides and cholesterol. Our results indicated that there was a significant decline in free fatty acids levels under metabolic stress conditions (Fig. 4a, Supplementary Fig. 4a, b). However, in the absence of caspase-10 no significant decline in free fatty acids levels was observed under metabolic stress conditions. Furthermore, upon simultaneous abrogation of ACLY expression, free fatty acids levels declined and there was no change upon metabolic stress. Concurring observations were made in other cell types including HCT116 and IMR90 (Supplementary Fig. 4c, d). Similar results were obtained when triglyceride and total cholesterol levels were analyzed (Fig. 4b, c). Caspase-10-mediated downregulation of free fatty acids levels was abrogated upon depletion of FADD but not upon caspase-3 depletion (Supplementary Fig. 4e). Moreover, in presence of cleavage-resistant mutant ACLY^D1026A^, no significant decline in free fatty acids, triglycerides and cholesterol levels was observed under metabolic stress conditions (Supplementary Fig. 4f-h). To examine total intracellular lipid content, we also performed Nile Red staining. We observed a significant decrease in intracellular lipid content under metabolic stress conditions (Fig. 4d). However, in absence of caspase-10, there was no significant change in intracellular lipid content upon metabolic stress. Taken together these results suggest that caspase-10-mediated cleavage of ACLY suppresses lipogenesis.

Previous studies suggest that ACLY-mediated regulation of acetyl-CoA levels is critical for the sustenance of global histone acetylation^12^. Thus, to further delineate the effect of caspase-10-mediated ACLY cleavage, we examined global histone acetylation status. We observed a significant decline in global acetylated levels of histones H3 and H4 concomitant to the activation of caspase-10 and ACLY cleavage at extended time points of metabolic stress (36 h and 48 h) (Fig. 4e, Supplementary Fig. 4i, j). However, no significant change was observed in H3 and H4 acetylation status in absence of caspase-10 over the time period of metabolic stress. Co-depletion of ACLY along with caspase-10 constitutively suppressed histones H3 and H4 acetylation over the time course of metabolic stress. Similar observations were made in other cell types including HCT116 and IMR90 (Supplementary Fig. 4k, l). These results indicate that caspase-10 could downregulate H3 and H4 acetylation in an ACLY-dependent manner. Furthermore, to examine whether the decline in histone acetylation was indeed due to decrease in acetyl-CoA levels, we supplemented cells with acetate, which is converted to acetyl-CoA by acetyl-CoA synthetase 2 (ACSS2)^25^. Supplementation with acetate under glucose starvation conditions restored the acetylation status of histones H3 and H4 (Fig. 4f). These results suggest that the decrease in histone acetylation was indeed due to reduced levels of acetyl-CoA. In addition, we observed that in presence of cleavage-resistant ACLY mutant (ACLY^D1026A^) there was no significant change in acetylation levels of histones H3 and H4 upon glucose starvation (Fig. 4g). Thus, these findings indicate that activation of caspase-10 under metabolic stress conditions and subsequent cleavage of ACLY results in the downregulation of acetyl-CoA levels and abrogates global acetylation of H3 and H4.

### Caspase-10 downregulates proliferative and metastatic genes

**Fig. 5:**
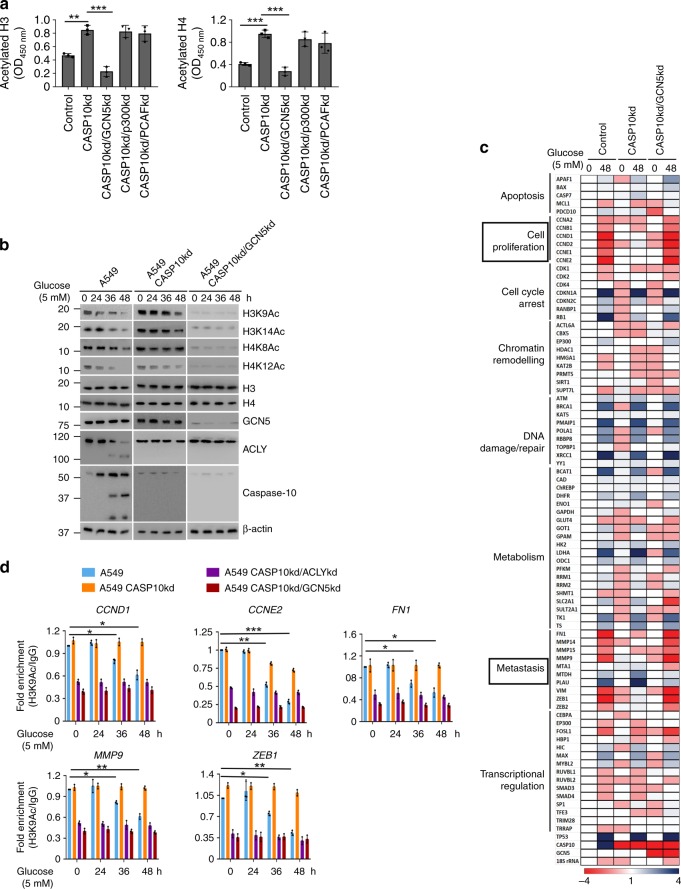
Caspase-10 represses proliferative and metastatic genes **a** A549 control (control), caspase-10 knockdown (CASP10kd), caspase-10/GCN5 double knockdown (CASP10kd/GCN5kd), caspase-10/p300 double knockdown (CASP10kd/p300kd) and caspase-10/PCAF double knockdown (CASP10kd/PCAFkd) cells were subjected to glucose starvation for 48 h. The cells were then harvested and sandwich ELISA was performed for acetylated H3 and acetylated H4. Error bars are means ± SD of biological replicates. Statistical analyses were done using one-way ANOVA (Tukey’s post hoc test). **P < 0.01; ***P < 0.001. **b** A549 control (A549), caspase-10 knockdown (A549 CASP10kd) and caspase-10/GCN5 double knockdown (A549 CASP10kd/GCN5kd) cells were subjected to glucose starvation for the indicated time points. These cells were harvested and western blotting was performed for the indicated proteins. **c** A549 control (control), caspase-10 knockdown (CASP10kd), caspase-10/ACLY double knockdown (CASP10kd/ACLYkd) and caspase-10/GCN5 double knockdown (CASP10kd/GCN5kd) cells were subjected to glucose starvation for the indicated time points. Total RNA was then isolated and relative mRNA levels were analyzed by RT-qPCR for the indicated genes. Heat map comparing relative mRNA levels (mean fold change from three independent experiments with triplicate samples) is shown. Blue and red indicate upregulation or downregulation respectively. **d** A549 control (A549), caspase-10 knockdown (A549 CASP10kd), caspase-10/ACLY double knockdown (A549 CASP10kd/ACLYkd) and caspase-10/GCN5 double knockdown (A549 CASP10kd/GCN5kd) cells were subjected to glucose starvation for the indicated time points. ChIP assay was then performed with control IgG or acetylated-H3K9 antibody. Error bars are means ± SD of three biological replicates. Statistical analyses were done using two-way ANOVA (Tukey’s post hoc test). *P < 0.05; **P < 0.01; ***P < 0.001. Source data are provided as a Source Data file

To gain the mechanistic insights into the alteration of histone H3 and H4 acetylation status, we next sought to identify the histone acetyltransferase involved. For this purpose, we co-depleted caspase-10 along with each of the three key HATs viz. PCAF, GCN5 and p300. Under metabolic stress conditions, we observed increased histone H3 and H4 acetylation in absence of caspase-10. However, this increase in histone acetylation was abrogated upon co-depletion of GCN5. Furthermore, co-depletion of PCAF or p300 along with caspase-10 did not lead to any significant change in histone H3 and H4 acetylation status (Fig. 5a). To further delineate the role of GCN5 in caspase-10-mediated downregulation of histones H3 and H4 acetylation, we analyzed the acetylation status of previously reported GCN5-targeted H3 and H4 lysine residues (H3K9, H3K14, H4K8, and H4K12)^26^, over the time course of metabolic stress. We observed that concomitant to increase in caspase-10 activation and ACLY cleavage at extended time points of metabolic stress, acetylation at H3K9, H3K14, H4K8, and H4K12 residues also declined (Fig. 5b, Supplementary Fig. 5a, b). However, absence of caspase-10 did not result in any change in the acetylation status at these histones residues over the time periods of glucose starvation. Moreover, simultaneous knockdown of GCN5 along with caspase-10 constitutively repressed the acetylation at H3K9, H3K14, H4K8 and H4K12 residues. Similar observations were made in other cell types including HCT116 and IMR90 (Supplementary Fig. 5c, d). These results indicate that caspase-10 impairs GCN5 activity to alter global histone H3 and H4 acetylation.

GCN5 serves as a coactivator of various oncogenic transcription factors including Myc and E2F^27,28^. Thus, we examined the effect of caspase-10 on E2F and Myc target genes. We observed that targets of E2F and Myc involved in cell proliferation and metastasis were specifically responsive to the presence of caspase-10 and GCN5 (Fig. 5c). Concomitant to decline in GCN5 activity upon metabolic stress, the expression of these genes was downregulated. However, depletion of caspase-10 abrogated the downregulation of proliferative and metastatic genes under metabolic stress conditions. Furthermore, co-depletion of caspase-10 and GCN5 repressed the expression of proliferative and metastatic genes. Concurringly, both H3K9Ac and H3K14Ac marks were downregulated at the promoter region of these genes at extended periods of metabolic stress (36 h and 48 h) (Fig. 5d, Supplementary Fig. 6a). However, upon caspase-10 knockdown, we did not observe any significant change in the levels of H3K9Ac and H3K14Ac at the promoter of either proliferative (*CCND1, CCNE2*) or metastatic genes (*FN1, MMP9, ZEB1*) over the time course of metabolic stress. In addition, co-depletion of either ACLY or GCN5 along with caspase-10 downregulated the epigenetic marks at the promoters of these proliferative and metastatic genes. Ectopic expression of GCN5 but not catalytically inactive mutant (GCN5^Y621A/P622A^) under conditions of caspase-10 and GCN5 co-depletion, abrogated the decline in the levels of the epigenetic marks at the promoters of these proliferative and metastatic genes upon metabolic stress (Supplementary Fig. 6b-d). To further examine whether decrease in the expression of these genes was indeed due to caspase-10-dependent decline in acetyl-CoA levels, we performed acetate supplementation studies. Acetate supplementation under metabolic stress conditions, promoted the expression of *CCND1, CCNE2, FN1, MMP9*, and *ZEB1*, concomitant to upregulation of histone H3K9Ac and H3K14Ac marks (Supplementary Fig. 7a-e). However, upon GCN5 knockdown, effect of acetate supplementation was abrogated and there was no significant change in the expression of these genes upon metabolic stress. Similar observations were made in other cell types including HCT116 and IMR90 (Supplementary Fig. 7f, g). These findings suggest that caspase-10 acts as an epigenetic modulator of proliferative and metastatic genes in a GCN5-dependent manner by determining acetyl-CoA levels.

### Caspase-10 suppresses ACLY oncogenic functions

**Fig. 6:**
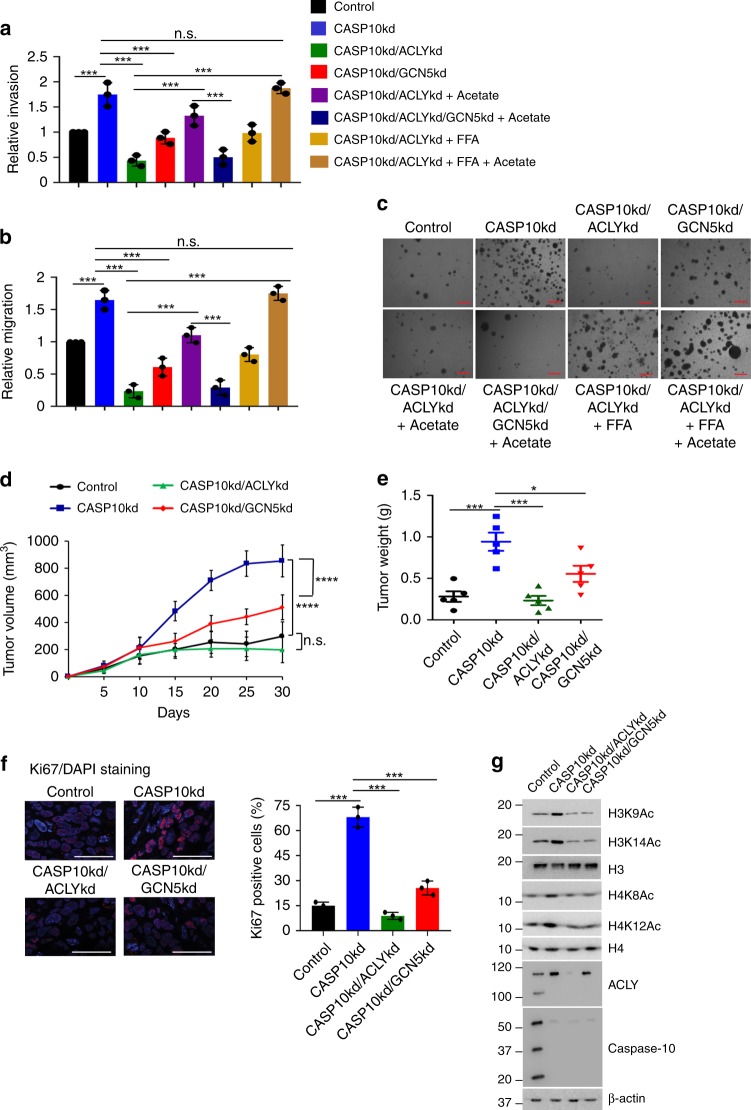
Caspase-10 represses ACLY promoted malignant phenotype **a**, **b** A549 control (control), caspase-10 knockdown (CASP10kd), caspase-10/ACLY double knockdown (CASP10kd/ACLYkd), caspase-10/GCN5 double knockdown (CASP10kd/GCN5kd), and caspase-10/ACLY/GCN5 triple knockdown (CASP10kd/ACLYkd/GCN5kd) cells were subjected to glucose starvation for 48 h. The cells were treated with sodium acetate (5 mM), palmitic acid (0.2 mM) or both for the last 24 h of starvation period as indicated. **a** In vitro invasion, and (**b**) migration potential of the cells was then measured. Error bars are means ± SD of three biological replicates. Statistical analyses were done using one-way ANOVA (Tukey’s post hoc test). ***P < 0.001. **c** A549 control, A549 CASP10kd, A549 CASP10kd/ACLYkd, A549 CASP10kd/GCN5kd, and A549 CASP10kd/ACLYkd/GCN5kd cells were seeded in soft agar and maintained in glucose starvation (5 mM) conditions, and treated with sodium acetate (5 mM), palmitic acid (0.2 mM) or both as indicated. Scale bars: 200μm. Representative images of colonies are shown. **d** A549 control (control), A549 CASP10kd, A549 CASP10kd/ACLYkd, and A549 CASP10kd/GCN5kd cells were injected subcutaneously into nude mice. Post-one week of injection, mice were treated with metformin (5 mg/ml in drinking water). Tumor volume was measured on the indicated days. The data shown are representative of three biological replicates (*n* = 5 mice/independent experiment). Error bars represent mean ± SD from five individual mice. Statistical analyses were done using one-way ANOVA (Tukey’s post hoc test). ****P < 0.0001. **e** At the end of 30 days, tumors were excised and weighed. The data shown are representative of three biological replicates (*n* = 5 mice/independent experiment). Error bars represent mean ± SD from five individual mice. Statistical analyses were done using one-way ANOVA (Tukey’s post hoc test). *P < 0.05; ***P < 0.001. **f** Immunofluorescence staining for Ki67 was performed on tumor sections (panel **d** above). Results shown is the representative of staining in five individual biological samples performed in triplicates. Statistical analyses were done using one-way ANOVA (Tukey’s post hoc test). *P < 0.05; **P < 0.01; ***P < 0.001. Scale bar: 10 μm. **g** At the end of 30 days, lysates of tumors (panel **d** above) were analyzed by immunoblotting. The data shown are representative of three independent experiments. Source data are provided as a Source Data file

Since ACLY has been reported to be critical for cancer cell proliferation and metastasis^29–31^, we next examined the effect of caspase-10 on ACLY-promoted malignant phenotype. Our data suggest that under metabolic stress conditions, depletion of caspase-10 resulted in enhanced invasiveness and migration potential (Fig. 6a, b, Supplementary Fig. 8a, b). However, co-depletion of caspase-10 along with ACLY or GCN5 significantly reduced the invasiveness and migration potential. Furthermore, the decrease in the invasiveness and migration potential was more pronounced in A549 CASP10/ACLY double knockdown cells as compared to A549 CASP10/GCN5 double knockdown cells. This could be due to the fact that depletion of ACLY suppresses both lipogenesis and global histone acetylation, while depletion of GCN5 interferes only with the histone acetylation. Thus, to explore the contribution of alteration in histone acetylation and cellular lipids levels, we further examined the invasiveness and migration potential under conditions of acetate and free fatty acid (FFA) supplementation (Fig. 6a, b, Supplementary Fig. 8a, b). We observed that supplementation of acetate significantly elevated invasiveness and migration potential of A549 CASP10/ACLY double knockdown cells. However, this increase was not observed in A549 CASP10/ACLY/GCN5 triple knockdown cells. Moreover, supplementation of free fatty acid in A549 caspase-10/ACLY double knockdown cells resulted in higher invasiveness and migration potential as compared to unsupplemented A549 caspase-10/ACLY double knockdown cells but lower than cells supplemented with acetate. We also observed that upon supplementation of both acetate and free fatty acid in A549 caspase-10/ACLY double knockdown cells, the invasiveness and migration potential was restored to the levels of A549 caspase-10 knockdown cells. Furthermore, in presence of cleavage-resistant mutant ACLY^D1026A^ significantly higher invasiveness and migration potential was observed under metabolic stress conditions, as compared to wild-type ACLY (Supplementary Fig. 8c, d). Similar results were obtained with HCT116 cells (Supplementary Fig. 8e, f). Concurring observations were made when anchorage-independent growth was examined (Fig. 6c, Supplementary Fig. 8g). Taken together, these results indicate that caspase-10 represses ACLY-mediated malignant transformation.

We next investigated the effect of caspase-10 on ACLY-promoted tumorigenesis. We observed that under metabolic stress conditions, caspase-10 depletion results in significantly larger tumors, which was attenuated upon co-depletion of ACLY or GCN5 (Fig. 6d, e). Furthermore, increased Ki67 staining in tumor sections, which is indicative of higher proliferation rate, was observed upon caspase-10 knockdown, while simultaneous knockdown of ACLY or GCN5 significantly reduced the extent of staining (Fig. 6f). We also investigated the level of histone acetylation in these tumors. Our data suggest that depletion of caspase-10 results in elevated levels of H3K9, H3K14, H4K8, and H4K12 acetylation while co-depletion of ACLY or GCN5 downregulated these acetylation marks (Fig. 6g). However, caspase-10-mediated repression of ACLY-promoted tumorigenesis was abrogated upon depletion of FADD and to a lesser extent upon caspase-3 depletion (Supplementary Fig. 9a, b). We also examined the extent of lipogenesis in these tumors. Our results indicated that the levels of free fatty acids were significantly elevated in the absence of caspase-10 under metabolic stress conditions (Supplementary Fig. 9c). However, upon co-depletion of ACLY free fatty acids levels were downregulated while no significant change was observed upon GCN5 co-depletion. Similarly, upon caspase-3 depletion free fatty acids levels were downregulated while no significant change was observed upon FADD depletion. These results suggest that caspase-10 represses ACLY-mediated tumorigenesis through a mechanism involving both epigenetic and metabolic alterations.

### Caspase-10 inhibits ACLY-promoted aggressive tumor phenotype

**Fig. 7:**
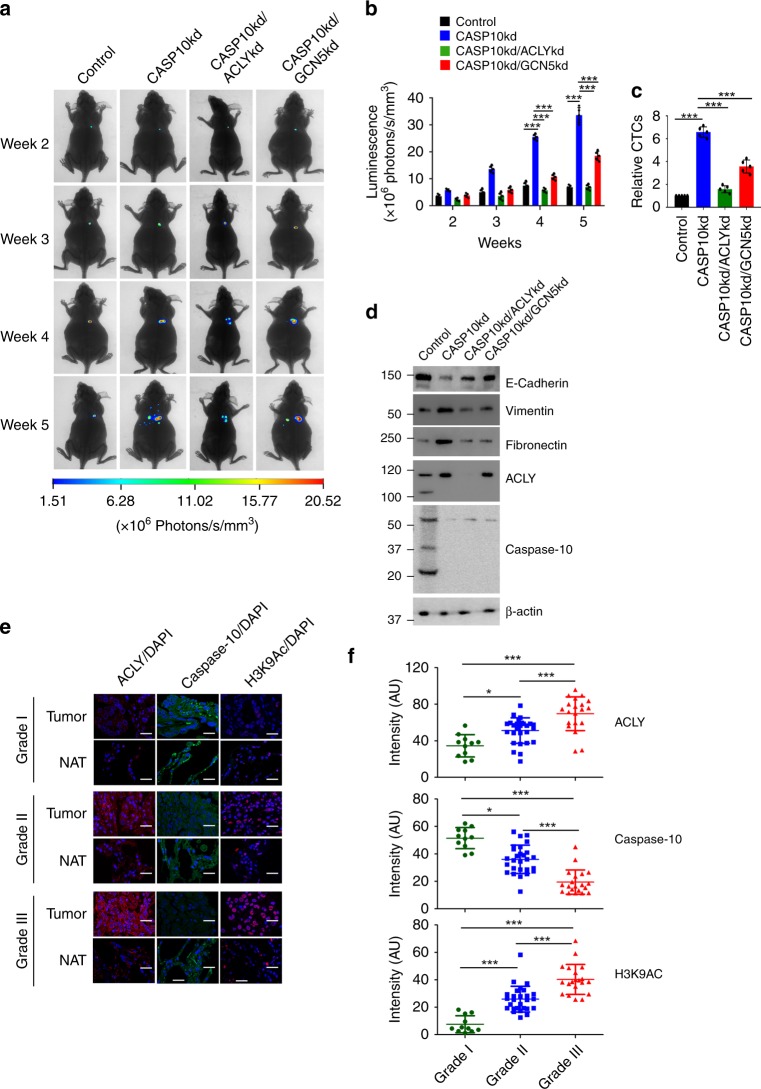
Caspase-10 downregulates ACLY-promoted aggressive tumor phenotype **a** A549^*Luc2*^ control (control), caspase-10 knockdown (CASP10kd), caspase-10/ACLY double knockdown (CASP10kd/ACLYkd), and caspase-10/GCN5 double knockdown (CASP10kd/GCN5kd) cells were orthotopically injected into the lung of nude mice. Post-one week of injection, mice were administered metformin (5 mg/ml in drinking water). Bioluminescence imaging was performed weekly and representative images are shown. The data shown are representative of three independent experiments using five individual mice per group. **b** Bioluminescence quantification (panel **a** above) was performed at indicated time points. The data shown are representative of three independent experiments using five individual mice per group. Error bars are mean ± SD from five individual mice (n = 5 mice per group). Statistical analyses were done using two-way ANOVA (Tukey’s post hoc test). ***P < 0.001. **c** At the end of 4 weeks, blood from mice in (panel **a** above), was used to isolate genomic DNA for examining circulating tumor cells. The data shown are representative of three independent experiments using five individual mice per group. Error bars are mean ± SD from five individual mice (*n* = 5 mice per group). Statistical analyses were done using one-way ANOVA (Tukey’s post hoc test). ***P < 0.001. **d** At the end of 5 weeks, tumor lysates were prepared (panel **a** above) and analyzed by immunoblotting for the indicated proteins. The data shown are representative of three independent experiments using five individual mice per group. **e** Representative imag**e** of immunostaining of ACLY, caspase-10 and H3K9Ac in different grades of human lung adenocarcinoma and matched normal adjacent tissue (NAT). DAPI was used to counter stain nucleus. Scale bar: 20 μm. **f** Quantitation of ACLY, caspase-10 and H3K9Ac levels in different grades of human lung adenocarcinoma with respect to matched normal adjacent tissue. The data shown are representative of three independent experiments. Error bars are mean ± SD. Statistical analyses were done using one-way ANOVA (Dunn’s multiple comparison test). *P < 0.05, ***P < 0.001. Source data are provided as a Source Data file

We next examined the effect of caspase-10 on ACLY promoted tumor malignancy in orthotopic lung cancer model. We observed that under metabolic stress conditions, caspase-10 knockdown cells formed much larger tumors as compared to control cells (Fig. 7a, b). Depletion of caspase-10 also resulted in numerous metastatic nodules in the liver (Supplementary Fig. 9d). We also observed higher number of circulating tumor cells (CTCs) in mice implanted with caspase-10 knockdown cells as compared to control cells (Fig. 7c). This was further corroborated by the observation that the primary orthotopic tumors exhibited downregulation of epithelial marker E-cadherin and upregulation of mesenchymal markers vimentin and fibronectin, which is suggestive of increased predisposition of these tumors to metastasize (Fig. 7d). However upon co-depletion of ACLY or GCN5, significantly smaller tumors and much reduced metastasis was observed. Taken together, our results demonstrate that caspase-10 downregulates ACLY promoted tumor metastasis.

Since elevated levels of ACLY have been reported in lung adenocarcinoma^16,32^, we next examined the levels of ACLY and caspase-10 in different grades of lung adenocarcinoma. We observed that consistent with increase in ACLY levels, GCN5-targeted H3K9 acetylation levels were also upregulated with increasing grades of lung adenocarcinoma as compared to matched normal adjacent tissue (Fig. 7e, f). Conversely, we found that the levels of caspase-10 were markedly reduced with increasing aggressiveness of lung adenocarcinoma. Thus our data suggests that dysregulation of caspase-10 expression and consequent upregulation of ACLY is critical for lung cancer progression.

## Discussion

Catalytic activation of caspases is a tightly-regulated process and requires homodimerization of initiator caspases^33^. Thus, adapter proteins which bind to the caspases play a key role in activation and execution of various downstream functions. cFLIP_L_, FADD, AK2, and p62 have been reported as mediators of caspase-10 activation^18–21^. FADD binds to death-effector-domain (DED) of caspase-10 via its death-domain and plays a key role in Fas signaling mediated extrinsic apoptosis^18^. Furthermore, FADD and AK2-mediated activation of caspase-10 was demonstrated under genotoxic stress conditions^20^. Our data suggests that among the mediators, FADD and AK2 are critical for the metabolic-stress-induced autocleavage and activation of caspase-10. However, our findings indicate that caspase-10 activation upon metabolic stress is independent of extrinsic pathway of apoptosis. Thus, our study establishes the mechanism of intracellular activation of caspase-10 upon metabolic stress.

The key to understand the function of a caspase lies in the identification of its substrates. However, limited substrate repertoire restricts the understanding of plausible functions of caspase-10. In vitro studies have demonstrated the ability of caspase-10 to process caspase-3 and caspase-7^1,34^. Caspase-10 has also been reported to cleave Bid to induce FasL-mediated apoptosis^18^. Studies from mammary gland involution suggest that caspase-10 cleaves insulin receptor substrates (IRS-1 and IRS-2) and restricts Insulin-like growth factor-1 (IGF-1)-mediated signaling critical for sustenance of mammary epithelial cells^35^. Here we report the characterization of ACLY as a bonafide caspase-10 substrate. Caspase-10-mediated ACLY cleavage inhibits metabolic and epigenetic reprogramming pivotal for tumorigenesis. Owing to the crucial role of ACLY in lipogenesis, ACLY is upregulated in non-alcoholic fatty liver disease (NAFLD)^36^. Thus ACLY has emerged as a key pharmacological target for treatment of NAFLD^37^. However, the role of caspase-10 in NAFLD has not yet been explored. Since our study suggests that caspase-10 can abrogate ACLY activity, it would be interesting to explore whether there is a link between caspase-10 and NAFLD.

Owing its role in the generation of acetyl-CoA, ACLY is a tightly regulated enzyme. At transcript level, ACLY expression is induced by lipogenic transcription factor SREBP1 via Akt signaling^15^. However, activity of ACLY is primarily regulated through post-translation modifications. PI3K/Akt has been reported to phosphorylate ACLY at Ser454 thereby augmenting its activity^16^. Acetylation of ACLY at Lys540, Lys546, and Lys554 by PCAF inhibits UBR4-mediated ubiquitylation at these sites and hence, stabilizes ACLY protein^31^. Stabilization of ACLY promotes de novo lipid synthesis, cell proliferation and tumor growth. However, little is known about post-translational modifications negatively regulating ACLY activity/levels. Similar to UBR4-mediated ubiquitylation, Cullin 3 containing E3 ligase complex has also been reported to ubiquitylate ACLY at Lys546 which inhibits lipid synthesis and tumor progression^38^. Here, we report that caspase-10 cleaves ACLY at D1026 site. Even though the site lies in the citryl-CoA ligase domain^39^, mutating D1026 to alanine inhibits cleavage but has no effect on ACLY activity. Thus our studies add to the different modes of regulation of ACLY in response to diverse stimuli.

Acetyl-CoA is the sole donor of acetyl-moiety for histone acetylations, critical for epigenetic regulation of gene expression^40^. Hence, critical concentration of acetyl-CoA has to be maintained to drive the proliferation and neoplastic transformation of cancer cells. Among the acetyl-CoA generating metabolic pathways, two of them have been reported to be critical for maintaining acetyl-CoA levels to support histone acetylation viz. glucose-dependent production of acetyl-CoA by ACLY and acetate-dependent generation of acetyl-CoA by ACSS2^12,25^. Our data suggest that ACLY boosts acetyl-CoA levels to promote GCN5-mediated epigenetic reprogramming of proliferative and metastatic genes to spur tumorigenesis. Consequently, caspase-10-mediated ACLY cleavage restricts acetyl-CoA pool and attenuates GCN5 transcriptional program. Furthermore, in mouse tumor models, metabolic stress-induced caspase-10 downregulates ACLY levels which results in reduced tumor progression and metastasis. Thus our findings highlight a key role for caspase-10 in determining cellular metabolic outcomes and epigenetic control of gene expression, with major implications for tumor progression and metastasis.

## Methods

### Cell lines, culture conditions, and transfection

A549, IMR90, H1299, HCT116, and 293 A cells were cultured in DMEM with fetal bovine serum (Invitrogen) and 100U/ml penicillin and 100 µg/ml streptomycin at 37 °C. All cell lines were obtained from ATCC. The cell lines were authenticated by ATCC and routinely tested for absence of mycoplasma contamination. Amplification and titration of recombinant adenoviruses was performed in 293 A cells. Cells were cultured to ~50–70% confluency followed by infection with recombinant adenovirus at a multiplicity of infection (MOI) of 10–20. For glucose starvation conditions, cells were cultured to ~ 50% confluency and then grown in glucose-free DMEM (Invitrogen) with dialyzed fetal bovine serum (Invitrogen) and 5 mM glucose (Sigma). For inducing serum deprivation, cells were cultured to ~50% confluency and then grown in serum-free DMEM (Invitrogen). Transfections were performed using Lipofectamine 3000 (Invitrogen) for IMR90 and Lipofectamine 2000 (Invitrogen) for other cell lines according to manufacturer’s instructions. In transient transfection experiments, plasmid DNA was kept constant with empty vector. Metformin and Q-AEVD-FMK were purchased from Biovision, CA, USA. Sodium acetate and palmitic acid was purchased from Sigma Aldrich, MO, USA.

### Plasmids and shRNA

FLAG-tagged and Myc-tagged caspase-10 (pCMV6-CASP10D) and FLAG-tagged ACLY (pCMV6-ACLY) constructs were purchased from Origene. pEBB Flag GCN5 was a gift from Ezra Burstein (Addgene plasmid # 74784). pAd-Track Flag GCN5 Y621A/P622A was a gift from Pere Puigserver (Addgene plasmid # 14425). Recombinant adenovirus expressing CASP10-HA-FLAG and CASP10^C401A^-HA-FLAG, were generated using adenoviral plasmids pAdTrack-CMV and pAdEasy-1^41^. Mutant CASP10^C401A^ (MCLAB) and mutants of ACLY (ACLY^D79A^, ACLY^D144A^, ACLY^D318A^, ACLY^D1026A^) were generated by performing site-directed mutagenesis (Mutagenex, USA). For in vitro binding assay, mutant CASP10 (CASP10^C401A^) was cloned in pET-28a(+) vector.

pBabe-U6-shRNA plasmid was used to express shRNA against human *p53* (GACTCCAGTGGTAATCTAC), scrambled for human *p53* (GCACAGCATCATAGGTCTT), *LacZ* (CCAACGTGACCTATCCCATTA), human *CASP8* (GGGTCATGCTCTATCAGAT), human *SQSTM1* (ACTGGACCCATCTGTCTTCAA), human *FLIP* (GCAGTCTGTTCAAGGAGCA), human *AK2* (ATGGTAGTGGAGCTCATTG), human *FADD* (ACGTCATATGTGATAATGT), human *CASP3* (CCTGAGATGGGTTTATGTATA).

psiRNA-DUO plasmid (Invivogen) which allows independent expression of two shRNAs was used to express shRNA against human *CASP10* (GTTGGCAGAACTGACATGTGA), scrambled for human *CASP10* (GGGTATGAAGCGAGTCTTACA), human *ACLY* (GCCTCAAGATACTATACATTT), scrambled for human *ACLY* (GACCTTTACATCTAGACAATT); human *GCN5L2* (GAAGCUGAUUGAGCGCAAA), scrambled for human *GCN5L2* (GCAAAGGCCGAAATATGGT); human *p300* (GCAGCTCAACCATCCACTA), human *PCAF* (CCACCAUGAGUGGUGUCUA). The shRNAs were designed against the 3′ UTR of the transcript and hence cannot target ectopically expressed genes.

### Sequential Immunoprecipitation and LC/MS-MS

Recombinant adenoviruses expressing FLAG and HA-tagged mutant caspase-10 (CASP10^C401A^) were used to infect H1299 cells. Adenovirus expressing GFP was used as a negative control. Twenty-four hours post-infection, whole cell extracts were prepared. Cell extracts were immunoprecipitated with FLAG agarose conjugated beads (Santa Cruz) and eluted with 3× FLAG peptide (Sigma). The eluate was subjected to a second immunoprecipitation using HA agarose conjugated beads (Santa Cruz) followed by elution with HA peptide (Sigma). The final eluate was resolved by SDS-PAGE and visualized by silver staining. The bands were cut from SDS-PAGE gel, fully trypsinized and analyzed by reverse-phase liquid chromatography-tandem mass spectrometry (LC-MS/MS) (Proteomics International, Australia). The MS data were processed using the comparative proteomics analysis software suite MASCOT.

### Western blot and immunoprecipitation

Cell extracts were prepared using lysis buffer (20 mM Tris-HCl at pH 7.4, 5 mM EDTA, 10 mM Na_4_P_2_O_7_, 100 mM NaF, 2 mM Na_3_VO_4_, 1% NP-40, 1 mM phenyl methylsulphonyl fluoride (PMSF), 1× Protease inhibitor cocktail (Roche)). SDS-PAGE was performed for equal amount of protein per sample followed by transfer to a PVDF membrane (Millipore, Billerica, MA, USA). The antibodies used were as follows: p53 (Santa Cruz, sc-98, 1:1000), caspase-10 (MBL, M059-3, 1:1000), caspase-8 (Cell Signaling Techonology, 4790, 1:2000), ACLY (Cell Signaling Techonology, 13390, 1:2000), H3 (Cell Signaling Techonology, 4499, 1:2000), H4 (Cell Signaling Techonology, 2935, 1:2000), H3K9Ac (Cell Signaling Techonology, 9649, 1:2000), H3K14Ac (Cell Signaling Techonology, 7627, 1:2000), H4K8Ac (Cell Signaling Techonology, 2594, 1:2000), H4K12Ac (Cell Signaling Techonology, 13944, 1:2000), GCN5 (Cell Signaling Techonology, 3305, 1:2000), PCAF (Cell Signaling Techonology, 3378, 1:2000), β-actin (Santa Cruz, sc-47778, 1:1000), FLAG tag (Santa Cruz, sc-807, 1:1000), HA tag (Santa Cruz, sc-805, 1:1000), Fibronectin (Santa Cruz, sc-8422, 1:1000), E-cadherin (Santa Cruz, sc-8426, 1:1000), Vimentin (Santa Cruz, sc-6260, 1:1000), FADD (Santa Cruz, sc-271520, 1:1000), AK2 (Santa Cruz, sc-374095, 1:1000), SHMT (Santa Cruz, sc-365203, 1:1000), ME2 (Santa Cruz, sc-514850, 1:1000), FASN (Santa Cruz, sc-48357, 1:1000), PKM1 (Cell Signaling Techonology, 7067, 1:2000), p300 (Santa Cruz, sc-585, 1:1000), AcH3 (Active Motif, 39139, 1:1000), AcH4 (Active Motif, 39243, 1:1000), caspase-3 (Santa Cruz, sc-7272, 1:1000). Imaging of western blots was performed using a UVP ChemiDoc-it imager equipped with VisionWorksLS software (v7.1; UVP). Uncropped and unprocessed scans of the western blots are included in the Supplementary Information and Source Data file.

Five hundred microgram of cell lysate was used to perform immunoprecipitations. The cell lysate was pre-cleared with normal IgG antibodies. The pre-cleared cell extract was then incubated with indicated antibodies. Protein-A or protein-G Agarose beads was used for pull-down. Subsequent western blots were performed as described above.

### In vitro binding assay

His-CASP10^mut^ was bacterially expressed and purified. Recombinant ACLY protein was procured from Sino Biological Inc. In vitro binding assay was then performed using Pierce His Protein Interaction Pull-Down Kit (ThermoFisher Scientific) following the manufacturer’s protocol.

### RT-qPCR

Trizol (Invitrogen) was used for total RNA extraction and cDNA was synthesized using iScript DNA synthesis kit (Bio-Rad), following the manufacturer’s instructions. Maxima SYBR Green mastermix (Fermentas) was used to carry out qPCR in an Eppendorf Real time PCR machine. 18S rRNA served as an internal control. RT-qPCR data was analyzed by the ∆∆Ct method. Primer sequences are listed in Supplementary Data 1.

### ChIP assay

Chromatin immunoprecipitations were performed using a kit following the instructions provided by the manufacturer (Millipore). Briefly, 1 × 10^7^ cells were fixed using formaldehyde and lysed in SDS Lysis Buffer. To obtain DNA fragments between 0.2–1 kb, cell lysates were subjected to sonication. Preclearing was performed using Protein A agarose/Salmon sperm DNA slurry, followed by incubation of the samples with control IgG (Santa Cruz), or the indicated antibody overnight at 4 °C. This was followed by incubation of the samples with fresh Protein A agarose/Salmon sperm DNA slurry for 2 h and elution of the precipitated chromatin complexes with elution buffer (1% SDS, 0.1 M NaHCO_3_). Four hours of incubation at 65 °C was performed to reverse the DNA-protein cross-links. qPCR was then performed to analyze the immunoprecipitated DNA. Antibodies used were as follows: H3K9Ac (Cell Signaling Technology, 9649, 1:50) and H3K14Ac (Cell Signaling Technology, 7627, 1:50).

The primers used for this analysis were: *CCND1* (Forward: AGGTGTGTTTCTCCCGGTTA, Reverse: TTCCTACCTTGACCAGTCGG), *CCNE2* (Forward: TGGAAGATCAAAGCCATCGG, Reverse: CTGCCAGCTGTAACACTTCT), *FN1* (Forward: CCCAAAGTTTGTTTCCTCAATGTTA, Reverse: ATGGCTTGATAAACTCCCAGC) *MMP9* (Forward: GGCCAGGGGGATCATTAGTT, Reverse: TCCCTTGGTCTGAAAGCCTC), *ZEB1* (Forward: CAAGCGGAACTTCTAGCCTC, Reverse: CGGAGAGAGGCTACCTGAC).

### Caspase-10 activity assay

Caspase-10 activity was assayed following manufacturer’s instructions (Biovision). Briefly, cells were resuspended in 50 µl of chilled cell lysis buffer and incubated on ice for 10 min. Post-incubation, cells were centrifuged for 1 min in a microcentrifuge (10,000 rcf) and supernatant (cytosolic extract) was transferred to a fresh tube, placed on ice. One hundred microgram protein was diluted to 50 µl in cell lysis buffer for each assay. Reaction was setup with 50 µl of 2× Reaction Buffer (containing 10 mM DTT) and 5 µl of the 4 mM AEVD-pNA colorimetric substrate to each sample in a microtiter plate. Post incubation at 37 °C for 1 h, absorbance was measured at 405 nm in a microtiter plate reader.

### In vitro ACLY cleavage assay

Recombinant ACLY (Sino Biological) was incubated with a dilution series of recombinant caspase-10 (Biovision) for 1 h at 37 °C in standard caspase-assay buffer (20 mM PIPES, 100 mM NaCl, 10% sucrose, 10 mM DTT, 0.05% CHAPS, pH 7.4)^34^. Caspase-10 was preincubated in 1 M sodium citrate buffer (1.6 M sodium citrate, 20 mM PIPES and 100 mM NaCl, pH 7.4.) for 30 min at 37 °C. The reaction was terminated by adding 1× SDS loading buffer and boiling at 95 °C for 5 min. SDS-PAGE was performed, followed by western blotting for ACLY.

### TUNEL assay

Apoptosis was detected using TUNEL Assay Kit (Promega) following the manufacturer’s protocol.

### ACLY activity assay

ACLY activity was measured following the malate dehydrogenase-coupled enzymatic method^42^. Whole-cell lysates were added at a 1:19 ratio to the reaction mixture containing 100 mM Tris-HCl (pH 8.7), 20 mM potassium citrate, 10 mM MgCl_2_, 10 mM DTT, 0.5 U/ml malate dehydrogenase, 0.33 mM CoA, 0.14 mM NADH, and 5 mM ATP. Change in absorbance at 340 nm in the absence of exogenous ATP was subtracted from change in the presence of ATP and was normalized to protein concentration to calculate specific ACLY activity.

### Acetyl-CoA quantification assay

Nucleocytosolic fractions were prepared using Nuclear/Cytosol Fractionation Kit (Biovision) and Mammalian Mitochondria Isolation Kit (Biovision), following the manufacturer’s instructions. Fractions were deproteinized following perchloric acid/KOH protocol. Acetyl-CoA in nuclear-cytoplasmic fractions were quantified using PicoProbe™ Acetyl CoA Fluorometric Assay Kit (Biovision) as per manufacturer’s instructions.

### Triglyceride quantification assay

Total triglyceride content was measured using Triglyceride Quantification Colorimetric/Fluorometric Kit (Biovision) following the manufacturer’s protocol.

### Total cholesterol quantification assay

Total cholesterol content was measured using Total Cholesterol and Cholesteryl Ester Colorimetric Assay Kit II (Biovision) as per the manufacturer’s protocol.

### Free fatty acid quantification assay

Free fatty acid content was measured using Free Fatty Acid Quantification Colorimetric/Fluorometric Kit (Biovision) as per the manufacturer’s protocol.

### Nile Red staining

Cells were washed with PBS, fixed in 4% paraformaldehyde and stained with Nile Red (5 μg/ml). The cells were then counterstained with DAPI and visualized using Zeiss ApoTome microscope. Images were analyzed with Zeiss Zen Blue Software.

### Acid extraction of histones

The cells were harvested in cold NIB buffer (15 mM Tris-HCL pH7.5, 60 mM KCl, 15 mM NaCl, 5 mM MgCl_2_, 1 mM CaCl_2_, 250 mM sucrose, 1 mM DTT, 10 mM sodium butyrate, 0.1% NP-40 and protease inhibitors)^43^. The nuclei were pelleted at 600 rcf for 5 min at 4 °C. The pellets were then resuspended in 0.4 N H_2_SO_4_ and incubated for 30 min at 4 °C. After centrifugation at 11,000 rcf for 10 min at 4 °C, histones were precipitated from the supernatant by addition of 20% tricholoracetic acid followed by centrifugation at 16,000 rcf for 10 min at 4 °C. The pellet was washed once with acetone containing 0.1% HCl, and finally with 100% acetone. Histone proteins were dried at room temperature and resuspended in water.

### Acetylated histone sandwich ELISA assay

Acetylated histone H3 and H4 was quantified by performing sandwich ELISA using PathScan Sandwich ELISA kit (Cell Signaling Technology) following manufacturer’s protocol.

### Transwell migration assay

Cell migration was determined by Cultrex^®^cell migration assay (Trevigen, Gaithersburg, MD, USA). The cells were loaded in the upper chamber of 24-well transwell plate while DMEM medium with 10% FBS was present in lower chamber. Cell dissociation solution with 1 µM of Calcein-AM was used to collect cells after 24 h. The plate was read using 485 nm excitation and 520 nm emission filters and standard curves established for respective cell lines were used to calculate percentages of migrated cells.

### Transwell invasion assay

Cell invasion across basement membranes was measured using CultreCoat BME-coated cell invasion assay (Trevigen). Membranes were first rehydrated and then the protocol mentioned for the migration assay above was followed.

### Soft agar assay

The cells were suspended in medium containing 0.4% agar and overlaid on 1% agar in 6-well culture plates. The assay plates were incubated for 3 weeks. The colonies were counted and imaged.

### Xenograft studies

Institutional Animal Care and Use Committee of National Institute of Immunology, New Delhi, India approved all the animal studies. The right flank of female nude mice (nu/nu) was subcutaneously injected with 4 × 10^6^ cells/0.15 ml of DMEM. Post one week of injection, the mice were administered metformin (5 mg/ml) in the drinking water. The tumor size was evaluated for 30 days. Tumor volume was measured with a caliper and calculated using the formula: (widest diameter × smallest diameter^2^)/2. Post 30 days of injection, the mice were euthanized using carbon dioxide, followed by tumor excision and its weight determination. Tumor extracts were then prepared and immunoblotting was performed as described earlier. For Ki67 staining, tumors were fixed in formalin, washed with PBS, and embedded in paraffin for sectioning. Five micrometer sections were dewaxed and immunostained with Ki-67 antibody (Santa Cruz). Secondary detection was performed using anti-mouse Alexa Fluor 555 (Molecular Probes). Counterstaining was done with DAPI. The slides were imaged using a Zeiss ApoTome microscope and images were analyzed with the Zen Blue software.

### In vivo metastasis assay

A549 cells were stably transfected (pooled neomycin-resistant population) with pGL4.51[*luc2*/CMV/Neo] plasmid (Promega) to generate A549^*Luc2*^ cells. The cell suspension in DMEM containing 10% Matrigel at a density of 2 × 10^6^ cells/0.1 ml was injected intratracheally into the lungs of female nude mice. Post one week of injection, mice were administered metformin (5 mg/ml) in the drinking water. Tumors were allowed to develop for 5 weeks. For weekly luciferase imaging, mice were anaesthetized using Ketamine (80 mg/Kg) and Xylaxine (10 mg/Kg) by intraperitoneal injection. Anaesthetized mice were intraperitoneally injected D-luciferin (150 mg/Kg). Imaging was performed using Kodak imaging system FX Pro and images were analyzed using Carestream imaging software. Post 5 weeks, the mice were euthanized. Primary tumors were excised and western blot analysis was performed as described earlier. The liver was harvested and ex vivo imaging was performed.

Genomic DNA was isolated from blood obtained from euthanized mice for analyzing circulating tumor cells using the DNeasy Blood and Tissue Kit (Qiagen). qPCR was performed using primers specific to the human *Alu* repeat sequence (Forward: ACGCCTGTAATCCCAGCACTT, Reverse: TCGCCCAGGCTGGAGTGC), while mouse *actin* served as the control (Forward: GCTTCTTTGCAGCTCCTTCGTTG, Reverse: TTTGCACATGCCGGAGCCGTTGT).

### Tissue microarrays

Human lung adenocarcinoma and matched normal adjacent tissue sections were procured from US Biomax as tissue microarrays. For performing, immunofluorescence the paraffin sections were dewaxed and incubated with 1:100 caspase-10 antibody (MBL, M059-3), 1:100 ACLY antibody (Cell Signaling Technology, 13390) or 1:100 H3K9Ac antibody (Cell Signaling Technology, 9649). Anti-mouse Alexa Fluor 488 (Molecular Probes, A11001, 1:100) or anti-rabbit Alexa Fluor 555 antibody (Molecular Probes, A21428, 1:100) was used for secondary detection. Slides were mounted with with ProLong Gold antifade reagent with DAPI and imaging was performed using Zeiss Apotome microscope. The images were then analyzed using Zeiss Zen Blue software and the ImageJ software was used for quantitation. The average signal intensity (in arbitrary units, AU) from four random fields was used for the analysis.

### Statistical analysis

The number of independent experiments performed is reported in the figure legends. Results were expressed as individual data points and means ± SD. Statistical analyses of experiments were performed by a standard two-tailed Student’s *t*-test, one-way ANOVA and two-way ANOVA with Tukey’s, Bonferroni or Dunn’s multiple comparison test (indicated in figure legends) using Prism 8.0 (GraphPad). *P* < 0.05 was considered significant.

### Reporting summary

Further information on research design is available in the Nature Research Reporting Summary linked to this article.

## Supplementary information

Supplementary Information

Supplementary Data 1

Reporting Summary

Source data file

## Data Availability

The source data underlying Figs. 1–7 have been provided as a Source Data file. All the other data supporting the findings of this study are available within the article, supplementary information file and from the corresponding author upon reasonable request.
