# Supplementary material for: Caspase-10 inhibits ATP-citrate lyase-mediated metabolic and epigenetic reprogramming to suppress tumorigenesis
Source: Nat Commun. 2019 Sep 18;10:4255. doi: 10.1038/s41467-019-12194-6 (PMC6751159; doi:10.1038/s41467-019-12194-6)
Supplement: Supplementary file 1 — Supplementary Information [file 41467_2019_12194_MOESM1_ESM.pdf]

Caspase-10 inhibits ATP-citrate lyase-mediated metabolic and epigenetic reprogramming to suppress tumorigenesis

Kumari et al.

Supplementary Information

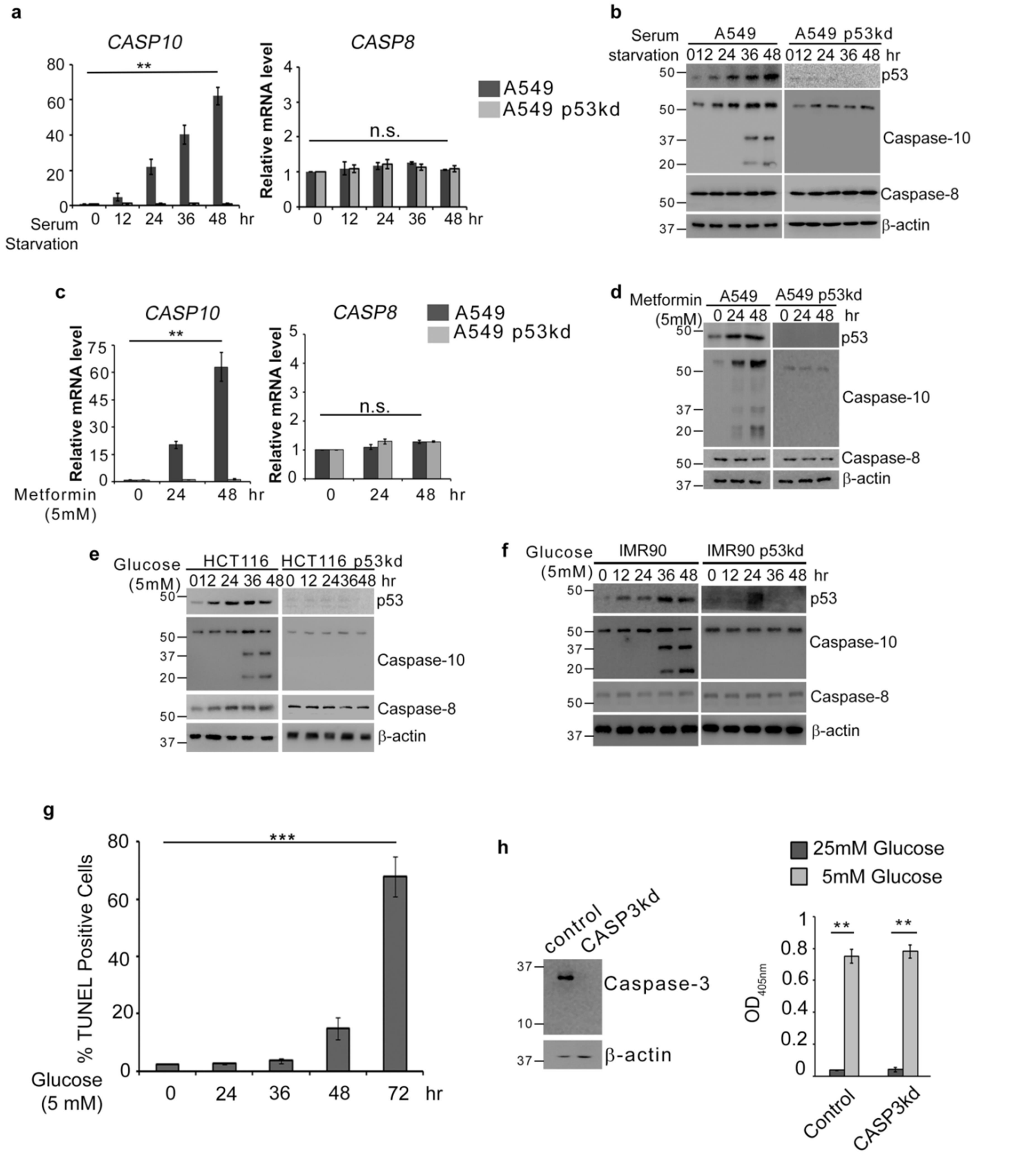

Supplementary Figure 1. Metabolic stress induces caspase-10 levels **a** A549 control (A549) and p53 knockdown (A549 p53kd) cells were subjected to serum starvation for indicated time points. The cells were then harvested and relative mRNA levels were analyzed. Error bars are means ± SD of three biological replicates. Statistical analyses were done using two-way

ANOVA (Tukey's post hoc test). **P<0.01. **b** A549 control and A549 p53kd cells were subjected to serum starvation for the indicated time points. The cells were then harvested and western blotting was performed. **c** A549 control (A549) and A549 p53 knockdown (A549 p53kd) cells were subjected to metformin (5mM) treatment for the indicated time points. The cells were then harvested and relative mRNA levels were analyzed. Error bars are means ± SD of three biological replicates. Statistical analyses were done using two-way ANOVA (Tukey's post hoc test). **P<0.01. **d** A549 control and A549 p53 knockdown (A549 p53kd) cells were subjected to metformin (5mM) treatment for the indicated time points. The cells were then harvested and western blotting was performed. **e** HCT116 control (HCT116) and p53 knockdown (HCT116 p53kd) cells were subjected to glucose starvation for indicated time points. The cells were harvested and western blotting was performed. **f** IMR90 control (IMR90) and p53 knockdown (IMR90 p53kd) cells were subjected to glucose starvation for indicated time points. The cells were harvested and western blotting was performed. **g** A549 cells were subjected to glucose starvation for indicated time points. The cells were then harvested and TUNEL assay was performed. Error bars are means ± SD of three biological replicates. Statistical analyses were done using unpaired t-test. ***P<0.001. **h** A549 control (control) and caspase-3 knockdown (CASP3kd) cells were harvested and western blotting was performed for the indicated proteins (top panel). A549 control (control) and caspase-3 knockdown (CASP3kd) cells were subjected to unstressed (25mM) or glucose starvation conditions (5mM) for 36 hours. The cells were then harvested and caspase-10 activity was examined (bottom panel). Error bars are means ± SD of three biological replicates. Statistical analyses were done using two-way ANOVA (Bonferroni's post hoc test). **P<0.01.

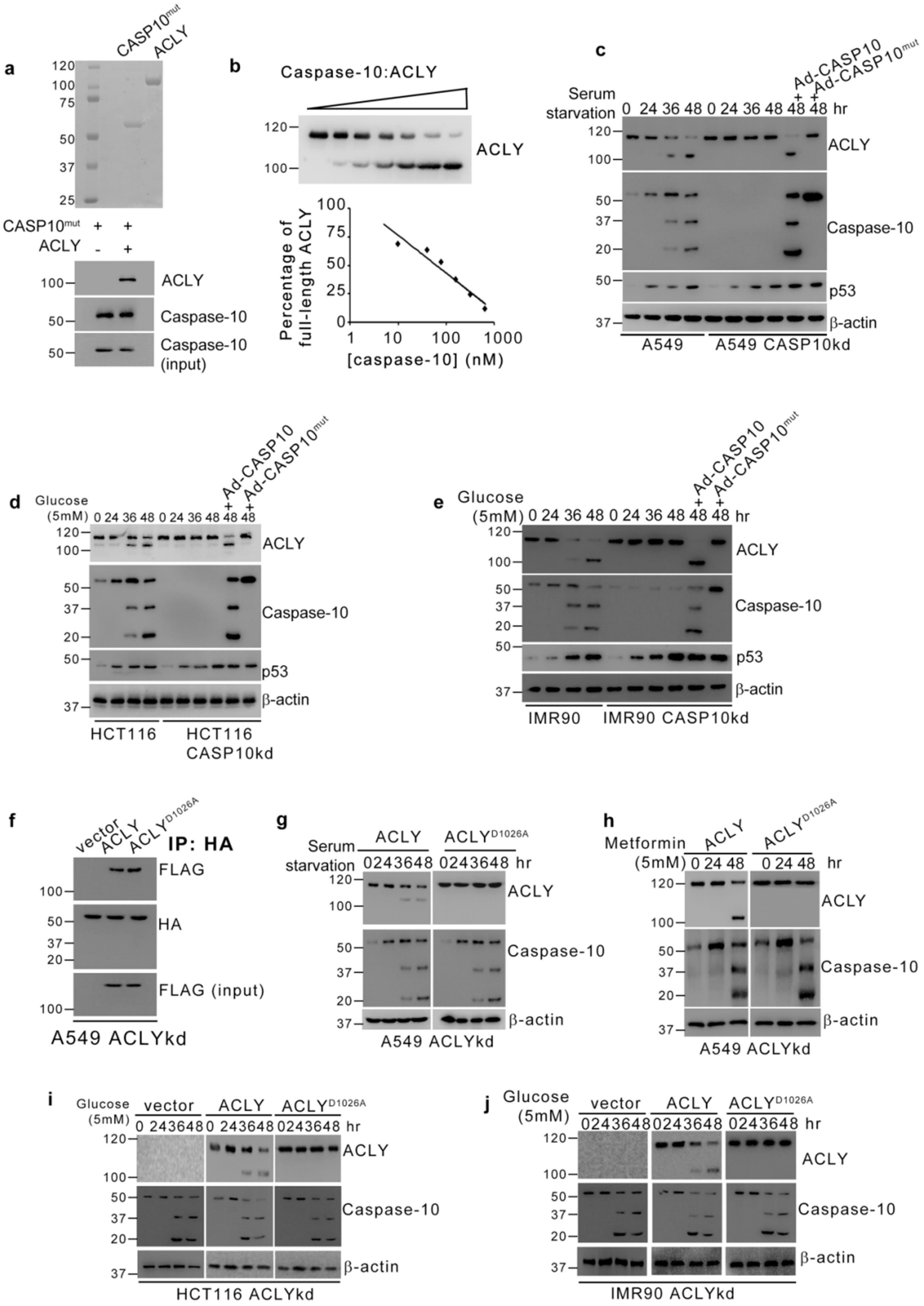

Supplementary Figure 2. ACLY is a caspase-10 substrate **a** His-CASP10^{mut} was bacterially expressed and purified. The indicated proteins were run on SDS-PAGE (top panel). *In vitro* binding assay was performed followed by western blotting (bottom panel). **b** *In vitro* ACLY cleavage assay was performed. **c** A549 control and CASP10kd cells were subjected to serum starvation for indicated time points. A549 CASP10kd cells were infected with Ad-CASP10 or

Ad-CASP10^{mut} during the last 12 hours prior to the end of the 48 hour time point. The cells were harvested and western blotting was performed. **d** HCT116 control and CASP10kd cells were subjected to glucose starvation for indicated time points. HCT116 CASP10kd cells were infected with Ad-CASP10 or Ad-CASP10^{mut} during the last 12 hours prior to the end of the 48 hour time point. The cells were harvested and western blotting was performed. **e** IMR90 control and CASP10kd cells were subjected to glucose starvation for indicated time points. IMR90 CASP10kd cells were infected with Ad-CASP10 or Ad-CASP10^{mut} during the last 12 hours prior to the end of the 48 hour time point. The cells were harvested and western blotting was performed. **f** A549 ACLYkd cells were co-transfected with HA-tagged caspase-10, and empty vector, FLAG-tagged wild-type (ACLY) or mutant ACLY (ACLY^{D1026A}), and treated with caspase-10 inhibitor (Q-AEVD-FMK) (25μM). 24 hours post-transfection, the cells were harvested and subjected to immunoprecipitation. **g** A549 ACLYkd cells were stably transfected with wild-type (ACLY) or mutant ACLY (ACLY^{D1026A}). These cells were subjected to serum starvation for indicated time points followed by western blotting. **h** A549 ACLYkd cells were stably transfected with wild-type (ACLY) or mutant ACLY (ACLY^{D1026A}). These cells were then subjected to metformin treatment for indicated time points followed by western blotting. **i** HCT116 ACLYkd cells were stably transfected with empty vector, wild-type (ACLY) or mutant ACLY (ACLY^{D1026A}). These cells were subjected to glucose starvation for indicated time points followed by western blotting. **j** IMR90 ACLYkd cells were stably transfected with empty vector, wild-type (ACLY) or mutant ACLY (ACLY^{D1026A}). These cells were subjected to glucose starvation for indicated time points followed by western blotting.

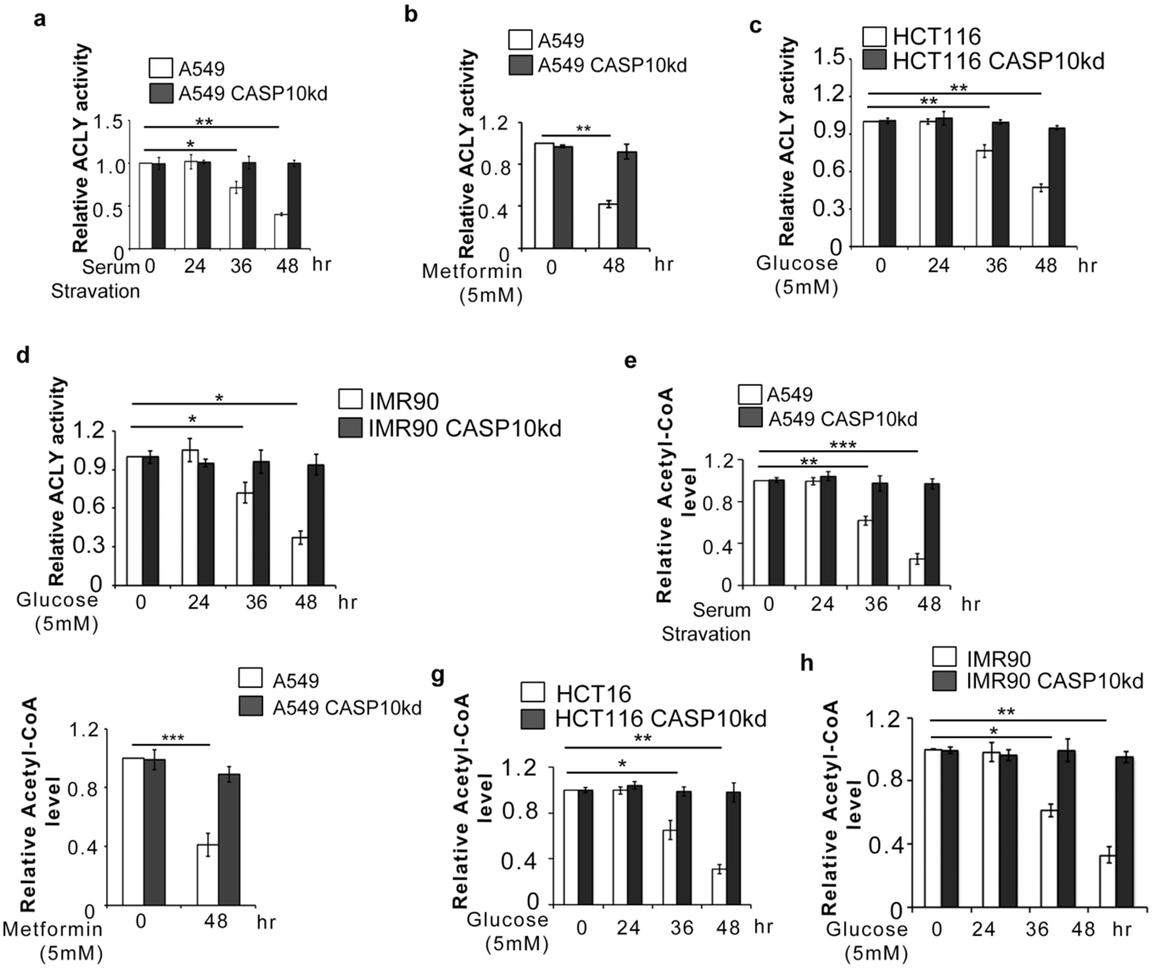

Supplementary Figure 3. Caspase-10 plays a key role in maintenance of acetyl-CoA levels **a** A549 control and A549 CASP10kd cells were subjected to serum starvation for indicated time points. ACLY activity was examined. Error bars are means ± SD of three biological replicates. Statistical analyses were done using two-way ANOVA (Bonferroni's post hoc test). **P*<0.05, ***P*<0.01. **b** A549 control and A549 CASP10kd cells were subjected to metformin treatment for indicated time points. ACLY activity was examined. Error bars are means ± SD of three biological replicates. Statistical analyses were done using two-way ANOVA (Bonferroni's post hoc test). ***P*<0.01. **c** HCT116 control and HCT116 CASP10kd cells were subjected to glucose starvation for indicated time points. ACLY activity was examined. Error bars are means ± SD of three biological replicates. Statistical analyses were done using two-way ANOVA (Bonferroni's post hoc test). ***P*<0.01. **d** IMR90 control and IMR90 CASP10kd cells were subjected to glucose starvation for indicated time points. ACLY activity was examined. Error

bars are means ± SD of three biological replicates. Statistical analyses were done using two-way ANOVA (Bonferroni's post hoc test). **P*<0.05. **e** A549 control and A549 CASP10kd cells were subjected to serum starvation for indicated time points. Acetyl-CoA levels were quantified. Error bars are means ± SD of three biological replicates. Statistical analyses were done using two-way ANOVA (Bonferroni's post hoc test). ***P*<0.01, ****P*<0.001. **f** A549 control and A549 CASP10kd cells were subjected to metformin treatment for indicated time points. Acetyl-CoA levels were quantified. Error bars are means ± SD of three biological replicates. Statistical analyses were done using two-way ANOVA (Bonferroni's post hoc test). ****P*<0.001. **g** HCT116 control and CASP10kd cells were subjected to glucose starvation for indicated time points. Acetyl-CoA levels were quantified. Error bars are means ± SD of three biological replicates. Statistical analyses were done using two-way ANOVA (Bonferroni's post hoc test). **P*<0.05, ***P*<0.01. **h** IMR90 control and CASP10kd cells were subjected to glucose starvation for indicated time points. Acetyl-CoA levels were quantified. Error bars are means ± SD of three biological replicates. Statistical analyses were done using two-way ANOVA (Bonferroni's post hoc test). **P*<0.05, ***P*<0.01.

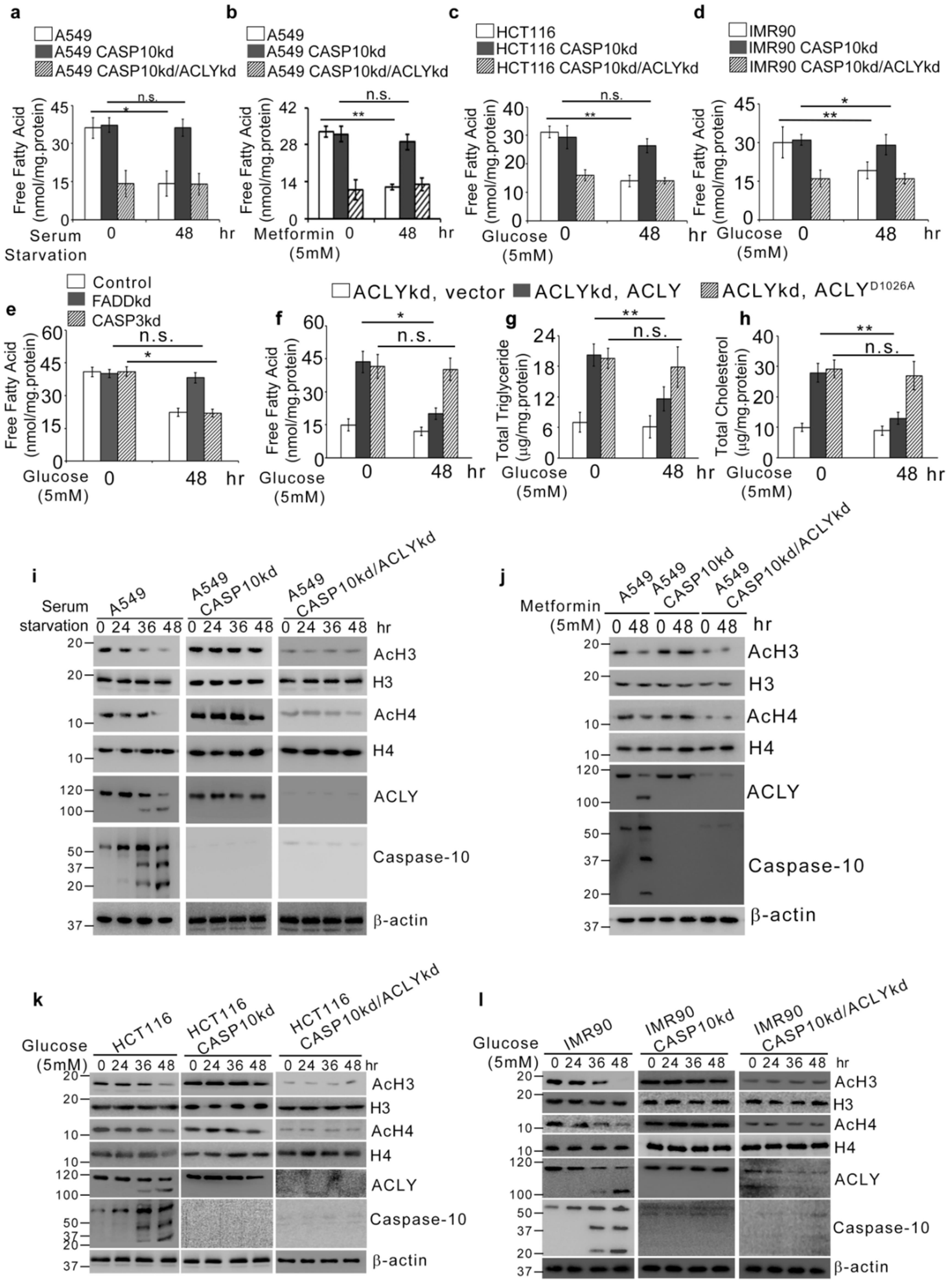

Supplementary Figure 4. Caspase-10 determines intracellular lipid levels and histone acetylation **a** A549 control, CASP10kd and CASP10kd/ACLYkd cells were subjected to serum starvation as indicated followed by free fatty acids quantification. Error bars are means ± SD of

three biological replicates. Statistical analyses were done using two-way ANOVA (Bonferroni's post hoc test). *P<0.05. **b** A549 control, CASP10kd and CASP10kd/ACLYkd cells were subjected to metformin treatment as indicated followed by free fatty acids quantification. Error bars are means ± SD of three biological replicates. Statistical analyses were done using two-way ANOVA (Bonferroni's post hoc test). **P<0.01. **c** HCT116 control, CASP10kd and CASP10kd/ACLYkd cells were subjected to glucose starvation as indicated followed by free fatty acids quantification. Error bars are means ± SD of three biological replicates. Statistical analyses were done using two-way ANOVA (Bonferroni's post hoc test). **P<0.01. **d** IMR90 control, CASP10kd and CASP10kd/ACLYkd cells were subjected to glucose starvation as indicated followed by free fatty acids quantification. Error bars are means ± SD of three biological replicates. Statistical analyses were done using two-way ANOVA (Bonferroni's post hoc test). *P<0.05, **P<0.01. **e** A549 control, Caspase-3kd and FADDkd cells were subjected to glucose starvation as the indicated followed by FFA quantification. Error bars are means ± SD of three biological replicates. Statistical analyses were done using two-way ANOVA (Bonferroni's post hoc test). *P<0.05. **f-h** A549 ACLY knockdown cells expressing empty vector, wild-type (ACLY) or mutant ACLY (ACLY^{D1026A}) were subjected to glucose starvation as indicated followed by (**f**) FFA, (**g**) total triglycerides, and (**h**) total cholesterol quantification. Error bars are means ± SD of biological replicates. Statistical analyses were done using two-way ANOVA (Bonferroni's post hoc test). *P<0.05, **P<0.01. **i** A549 control, CASP10kd and CASP10kd/ACLYkd cells were subjected to serum starvation as indicated. Western blotting was performed. **j** A549 control, CASP10kd and CASP10kd/ACLYkd cells were subjected to metformin treatment as indicated. Western blotting was performed. **k** HCT116 control, CASP10kd and CASP10kd/ACLYkd cells were subjected to glucose starvation as indicated. Western blotting was performed. **l** IMR90 control, CASP10kd and CASP10kd/ACLYkd cells were subjected to glucose starvation as indicated. Western blotting was performed.

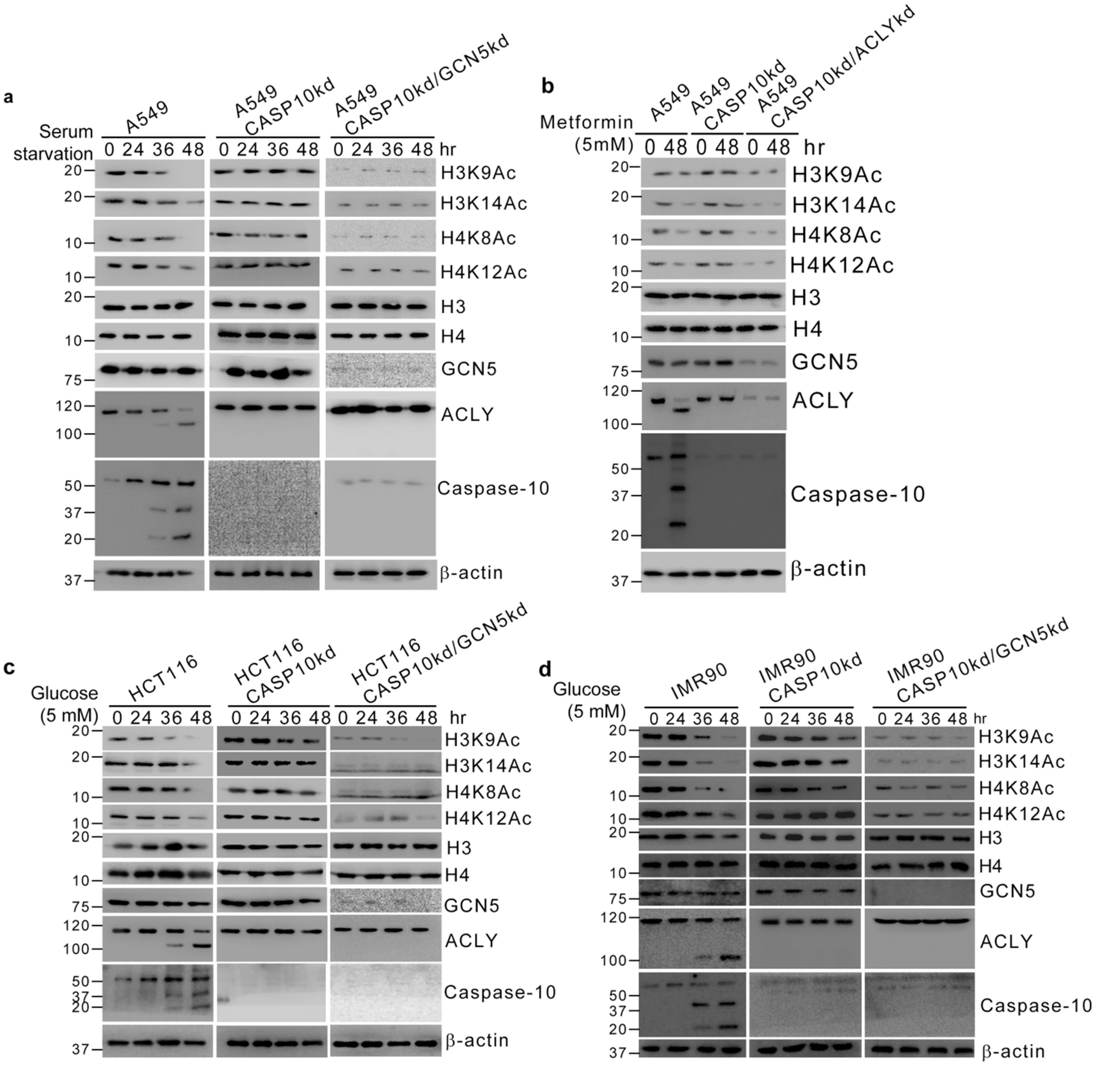

Supplementary Figure 5. Caspase-10 alters global histone H3 and H4 acetylation **a** A549 control (A549), caspase-10 knockdown (A549 CASP10kd) and caspase-10/GCN5 double knockdown (A549 CASP10kd/GCN5kd) cells were subjected to serum starvation for indicated time points. These cells were harvested and western blotting was performed for indicated proteins. **b** A549 control (A549), caspase-10 knockdown (A549 CASP10kd) and caspase-10/GCN5 double knockdown (A549 CASP10kd/GCN5kd) cells were subjected to metformin treatment for indicated time points. These cells were harvested and western blotting was performed for indicated proteins. **c** HCT116 control (HCT116), caspase-10 knockdown

(HCT116 CASP10kd) and caspase-10/GCN5 double knockdown (HCT116 CASP10kd/GCN5kd) cells were subjected to glucose starvation for indicated time points. These cells were harvested and western blotting was performed for indicated proteins. **d** IMR90 control (IMR90), caspase-10 knockdown (IMR90 CASP10kd) and caspase-10/GCN5 double knockdown (IMR90 CASP10kd/GCN5kd) cells were subjected to glucose starvation for indicated time points. These cells were harvested and western blotting was performed for indicated proteins.

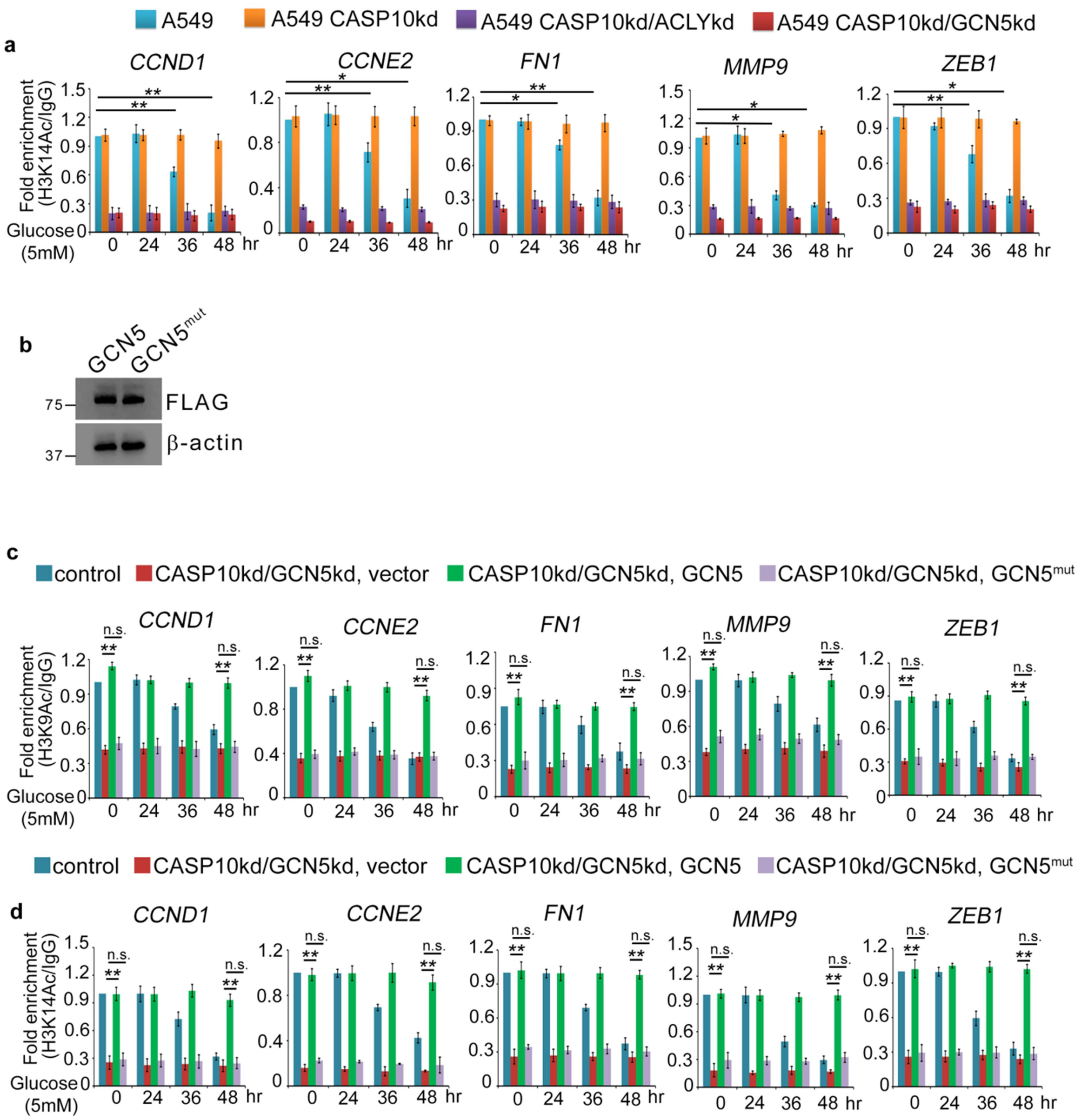

Supplementary Figure 6. Caspase-10 inhibits proliferative and metastatic genes expression **a** A549 control (A549), caspase-10 knockdown (A549 CASP10kd), caspase-10/ACLY double knockdown (A549 CASP10kd/ACLYkd) and caspase-10/GCN5 double knockdown (A549 CASP10kd/GCN5kd) cells were subjected to glucose starvation for the indicated time points. ChIP assay was then performed with control IgG or acetylated-H3K14 antibody. Error bars are means ± SD of three biological replicates. Statistical analyses were done using two-way ANOVA (Tukey's post hoc test). *P<0.05, **P<0.01. **b** A549 GCN5 knockdown cells were stably transfected (pooled hygromycin-resistant population) with wild-type or mutant GCN5.

The cells were harvested and western blotting was performed for indicated proteins. **c** A549 control (control), caspase-10/GCN5 double knockdown (CASP10kd/GCN5kd) cells as well as A549 caspase-10/GCN5 double knockdown cells expressing wild-type (CASP10kd/GCN5kd, GCN5) or mutant GCN5 (CASP10kd/GCN5kd, $GCN5^{mut}$) were subjected to glucose starvation for 48 hours. ChIP assay was then performed with control IgG or acetylated-H3K9 antibody. Error bars are means ± SD of three biological replicates. Statistical analyses were done using two-way ANOVA (Tukey's post hoc test). *P<0.05, **P<0.01. **d** A549 control (control), caspase-10/GCN5 double knockdown (CASP10kd/GCN5kd) cells as well as A549 caspase-10/GCN5 double knockdown cells expressing wild-type (CASP10kd/GCN5kd, GCN5) or mutant GCN5 (CASP10kd/GCN5kd, $GCN5^{mut}$) were subjected to glucose starvation for 48 hours. ChIP assay was then performed with control IgG or acetylated-H3K14 antibody. Error bars are means ± SD of three biological replicates. Statistical analyses were done using two-way ANOVA (Tukey's post hoc test). **P<0.01.

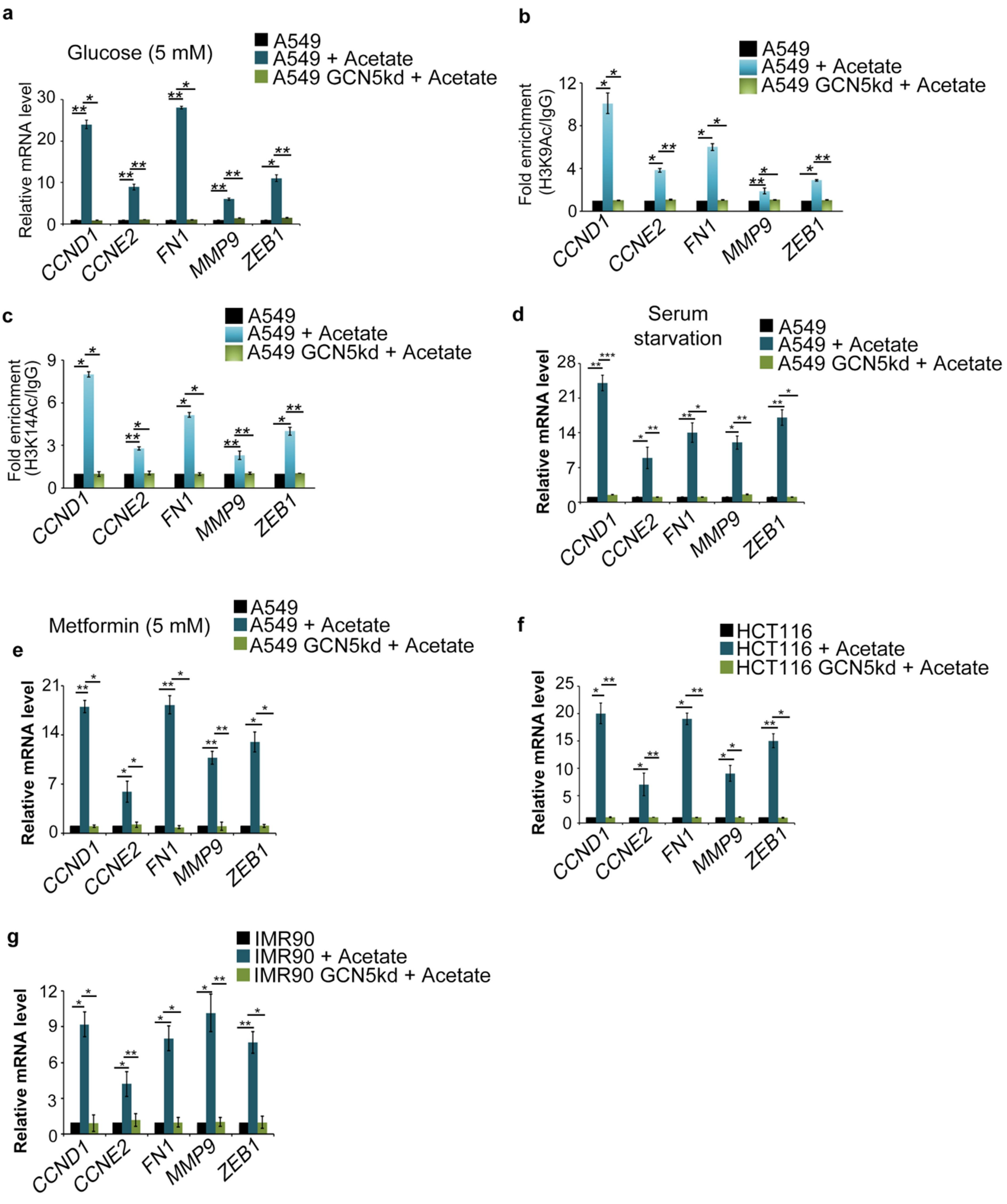

Supplementary Figure 7. Caspase-10 regulates epigenetic reprogramming of proliferative and metastatic genes **a** A549 control and GCN5kd cells were subjected to glucose starvation for 48 hours. The cells were treated with sodium acetate (5mM) for the last 24 hours of starvation period as indicated. RT-qPCR was then performed. Error bars are means ± SD of three biological

replicates. Statistical analyses were done using two-way ANOVA (Tukey's post hoc test). *P<0.05, **P<0.01. **b-c** A549 control and GCN5kd cells were subjected to glucose starvation for 48 hours. The cells were treated with sodium acetate (5mM) for the last 24 hours of starvation period as indicated. ChIP assay was then performed with indicated antibodies. Error bars are means ± SD of three biological replicates. Statistical analyses were done using two-way ANOVA (Tukey's post hoc test). *P<0.05, **P<0.01. **d-e** A549 control and GCN5kd cells were subjected to (**d**) serum starvation or (**e**) metformin treatment for 48 hours. Sodium acetate (5mM) was added to the cells for the last 24 hours of starvation period as indicated. RT-qPCR was then performed. Error bars are means ± SD of three biological replicates. Statistical analyses were done using two-way ANOVA (Tukey's post hoc test). *P<0.05, **P<0.01, ***P<0.001. **f** HCT116 control and GCN5kd cells were subjected to glucose starvation for 48 hours. The cells were treated with sodium acetate (5mM) for the last 24 hours of starvation period as indicated. RT-qPCR was then performed. Error bars are means ± SD of three biological replicates. Statistical analyses were done using two-way ANOVA (Tukey's post hoc test). *P<0.05, **P<0.01. **g** IMR90 control and GCN5kd cells were subjected to glucose starvation for 48 hours. The cells were treated with sodium acetate (5mM) for the last 24 hours of starvation period as indicated. RT-qPCR was then performed. Error bars are means ± SD of biological replicates. Statistical analyses were done using two-way ANOVA (Tukey's post hoc test). *P<0.05, **P<0.01.

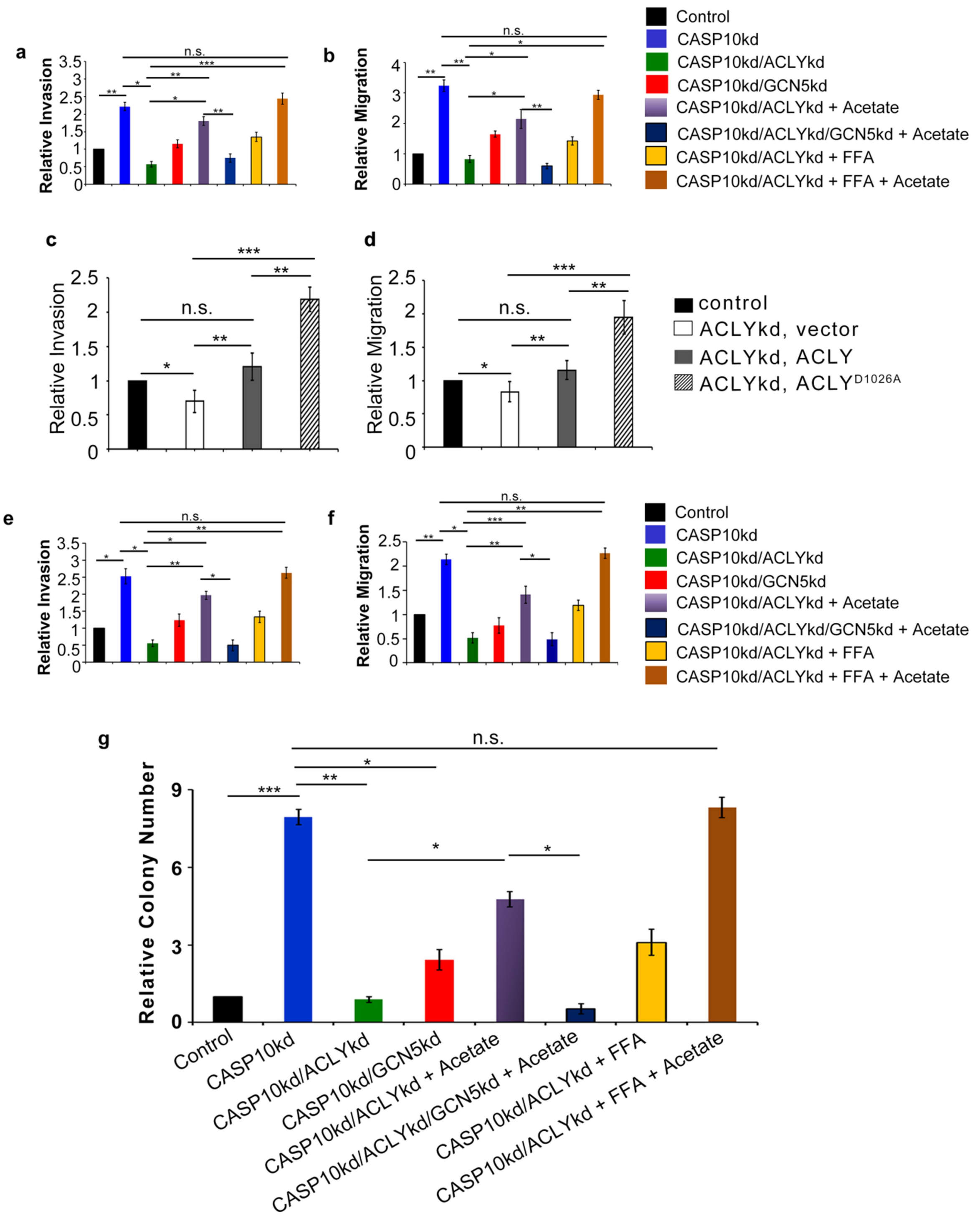

Supplementary Figure 8. ACLY oncogenic functions are repressed by caspase-10 **a-b** A549 control, CASP10kd, CASP10kd/ACLYkd, CASP10kd/GCN5kd, and CASP10kd/ACLYkd/GCN5kd cells were subjected to metformin treatment for 48 hours. Cells

were treated with sodium acetate (5mM), palmitic acid (0.2mM) or both for the last 24 hours of metformin treatment as indicated. (**a**) *In vitro* invasion, and (**b**) migration potential was then measured. Error bars are means ± SD of three biological replicates. Statistical analyses were done using one-way ANOVA (Tukey's post hoc test). *P<0.05, **P<0.01, ***P<0.001. **c-d** A549 ACLY knockdown cells expressing empty vector, wild-type (ACLY) or mutant ACLY ($ACLY^{D1026A}$) were subjected to glucose starvation for 48 hours. (**c**) *In vitro* invasion, and (**d**) migration potential was then measured. Error bars are means ± SD of three biological replicates. Statistical analyses were done using one-way ANOVA (Tukey's post hoc test). *P<0.05, **P<0.01, ***P<0.001. **e-f** HCT116 control, CASP10kd, CASP10kd/ACLYkd, CASP10kd/GCN5kd, and CASP10kd/ACLYkd/GCN5kd cells were subjected to glucose starvation for 48 hours. Cells were treated with sodium acetate (5mM), palmitic acid (0.2mM) or both for the last 24 hours of starvation period as indicated. (**e**) *In vitro* invasion, and (**f**) migration potential was then measured. Error bars are means ± SD of three biological replicates. Statistical analyses were done using one-way ANOVA (Tukey's post hoc test). *P<0.05, **P<0.01, ***P<0.001. **g** Enumeration of soft agar colonies (**Fig. 6c**). Numbers of colonies with a diameter greater than 20μm were counted. Error bars are means ± SD of three biological replicates. Statistical analyses were done using one-way ANOVA (Tukey's post hoc test). *P<0.05, **P<0.01, ***P<0.001.

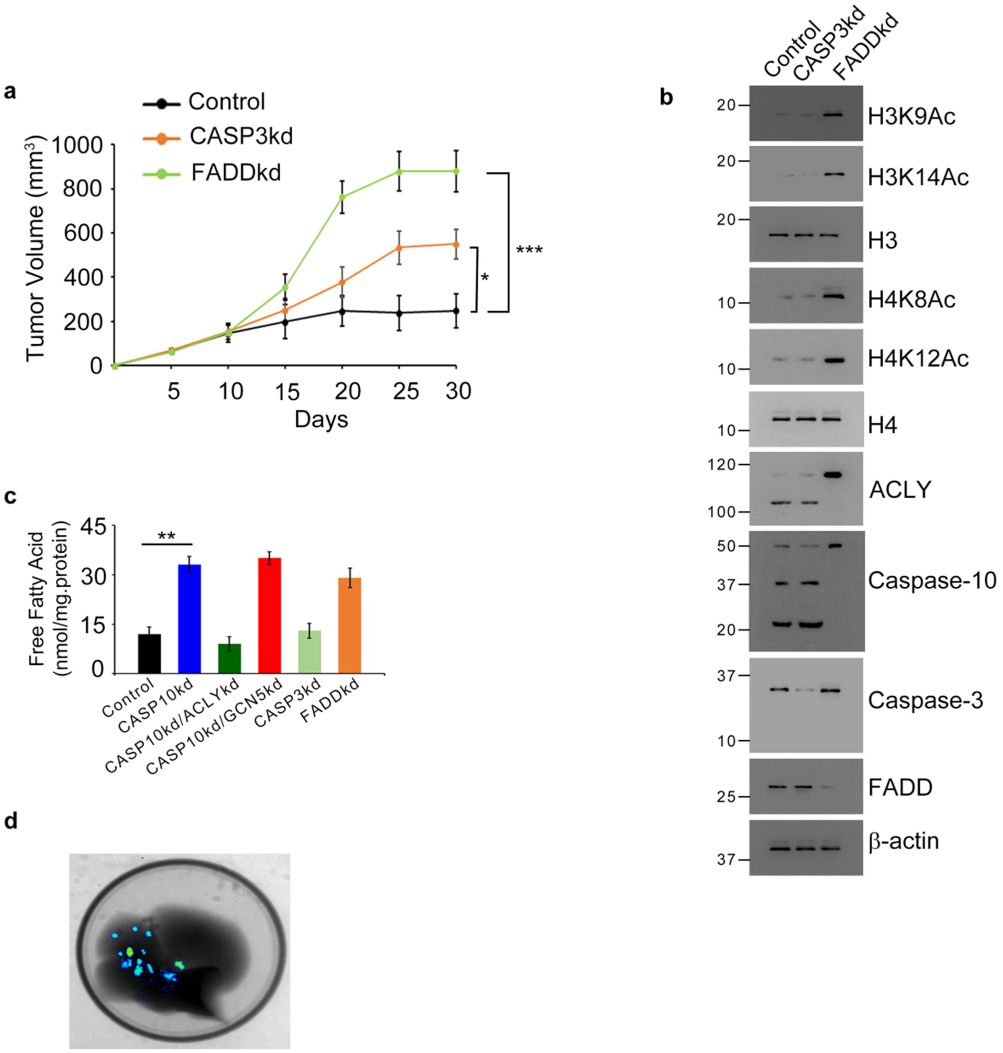

Supplementary Figure 9. Effect of caspase-3 and FADD on caspase-10 mediated repression of tumorigenesis **a** A549 control (control), caspase-3 knockdown (Caspase-3kd) and FADD knockdown (FADDkd) cells were injected subcutaneously into nude mice. Post-one week of injection, mice were treated with metformin (5mg/ml in drinking water). Tumor volume was measured on the indicated days. Error bars represent mean ± SD from five individual mice. The data shown are representative of three biological replicates (n = 5 mice/independent experiment). Statistical analyses were done using one-way ANOVA (Tukey's post hoc test). *P<0.05, ***P<0.001. **b** At the end of 30 days, lysates of tumors (**a**, above) were analyzed by immunoblotting for the indicated proteins. The data shown are representative of three independent experiments. **c** At the end of 30 days, lysates of tumors (From Fig. 6d and

Supplementary Fig. 9a) were subjected to free fatty acids quantification. The data shown are representative of three biological replicates (n = 5 mice/independent experiment). Statistical analyses were done using one-way ANOVA (Tukey's post hoc test). ***P*<0.01. **d** At the end of 5 weeks, liver was collected from the mice orthotopically implanted with $A549^{Luc2}$ caspase-10 knockdown cells (from **Fig. 7a**) and *ex vivo* imaging was performed. The data shown are representative of three independent experiments.

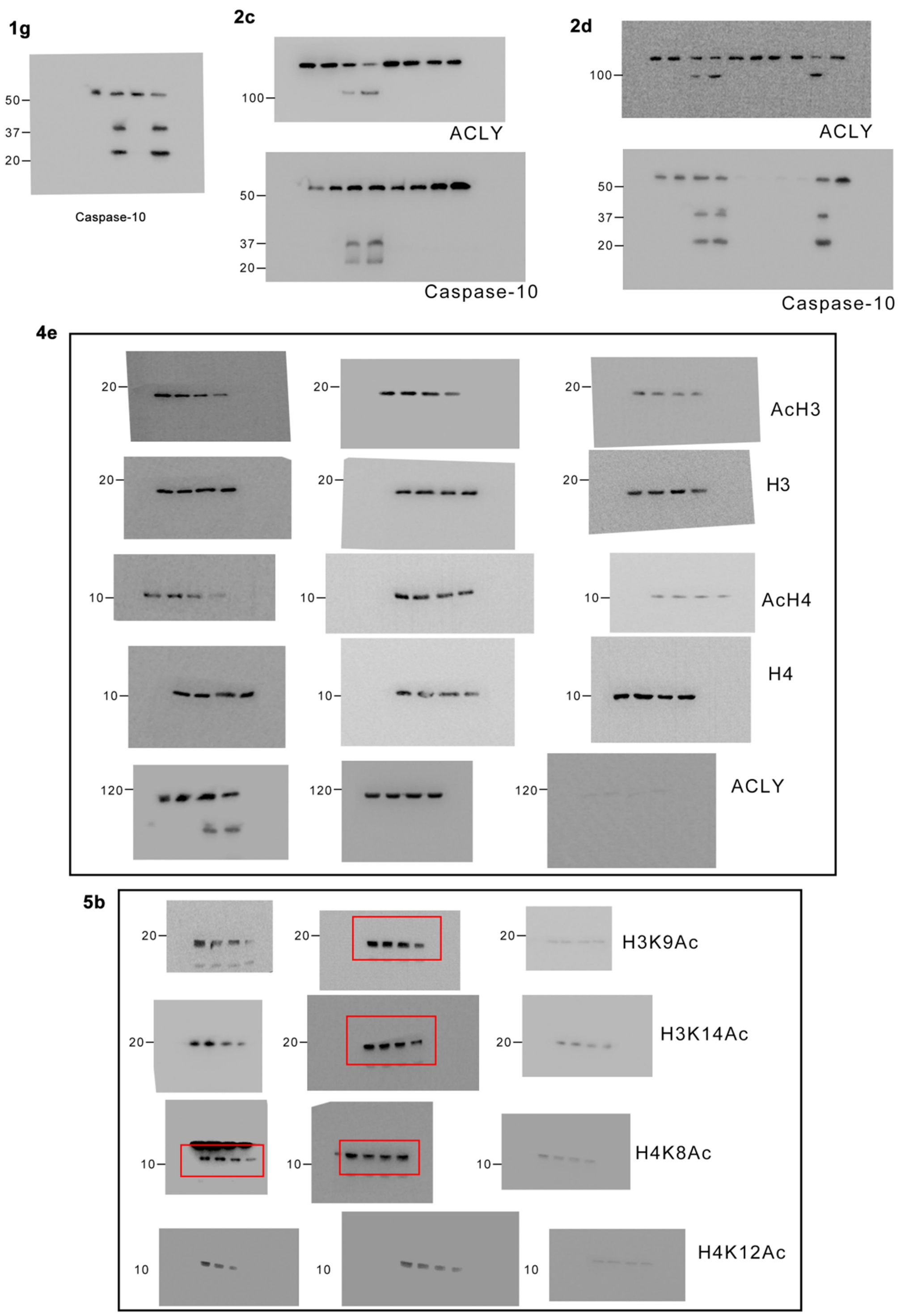

Supplementary Figure 10. Unprocessed scans of western blots
